# Cell Wall Composition as a Marker of the Reprogramming of the Cell Fate on the Example of a *Daucus carota* (L.) Hypocotyl in Which Somatic Embryogenesis Was Induced

**DOI:** 10.3390/ijms21218126

**Published:** 2020-10-30

**Authors:** Michał Kuczak, Ewa Kurczyńska

**Affiliations:** 1 Institute of Chemistry, Faculty of Science and Technology, University of Silesia in Katowice, 9 Szkolna St, 40–006 Katowice, Poland; mkuczak@us.edu.pl; 2 Institute of Biology, Biotechnology and Environmental Protection, Faculty of Natural Sciences, University of Silesia in Katowice, 28 Jagiellonska St, 40–032 Katowice, Poland

**Keywords:** cell fate reprogramming, cell wall, *Daucus carota*, formative division, pectins, AGPs, extensins, immunohistochemistry, pluripotency, totipotency

## Abstract

Changes in the composition of the cell walls are postulated to accompany changes in the cell’s fate. We check whether there is a relationship between the presence of selected pectic, arabinogalactan proteins (AGPs), and extensins epitopes and changes in cell reprogramming in order to answer the question of whether they can be markers accompanying changes of cell fate. Selected antibodies were used for spatio-temporal immunolocalization of wall components during the induction of somatic embryogenesis. Based on the obtained results, it can be concluded that (1) the LM6 (pectic), LM2 (AGPs) epitopes are positive markers, but the LM5, LM19 (pectic), JIM8, JIM13 (AGPs) epitopes are negative markers of cells reprogramming to the meristematic/pluripotent state; (2) the LM8 (pectic), JIM8, JIM13, LM2 (AGPs) and JIM11 (extensin) epitopes are positive markers, but LM6 (pectic) epitope is negative marker of cells undergoing detachment; (3) JIM4 (AGPs) is a positive marker, but LM5 (pectic), JIM8, JIM13, LM2 (AGPs) are negative markers for pericycle cells on the xylem pole; (4) LM19, LM20 (pectic), JIM13, LM2 (AGPs) are constitutive wall components, but LM6, LM8 (pectic), JIM4, JIM8, JIM16 (AGPs), JIM11, JIM12 and JIM20 (extensins) are not constitutive wall components; (5) the extensins do not contribute to the cell reprogramming.

## 1. Introduction

The formation of a multicellular organism from a single cell (zygote) requires the coordinated development of various cell types in a spatio-temporal manner [1]. During development, cells that have the same genetic information undergo differentiation. The concept of cell differentiation can be defined in two ways: (1) As a temporal process that focuses on the specialization in a structure and the function of a single cell, which is defined as cytodifferentiation, and (2) as the formation of the pattern(s), i.e., the appearance of heterogeneity in an initially homogeneous set of cells [2].

In vitro cultures are used as a model system that permits studies on the mechanisms that regulate cell differentiation, and the process of somatic embryogenesis (SE) is considered to be a good experimental system for analyzing this phenomena, including the reprogramming of cell fate [3]. SE is an unusual developmental process in which somatic cells, under appropriate conditions, produce cells that undergo a series of morphological and biochemical changes [3,4,5,6,7,8,9].

Cell walls are dynamic structures whose composition changes during plant growth, cell cytodifferentiation, and pattern formation [10,11]. Structural modifications, the reorganization of cell wall components, and the synthesis and insertion of new ones into existing walls are associated with changes in the tissue and organ morphology during plant growth [12,13]. The cell wall participates in cell adhesion, intercellular communication, protection of plants against pathogens, SE, and also determines the shape of cells [14,15,16].

Despite the diversity in the architecture of the cell walls, they are commonly built by polysaccharides such as cellulose, hemicelluloses and pectins, enzymes, and structural proteins such as hydroxyproline rich glycoproteins (HRGPs), which include the arabinogalactan proteins (AGPs), extensins, proline-rich proteins (PRPs) and glycine-rich proteins (GRPs) [17,18]. Immunohistochemical techniques [10] are one of the best methods to use to recognize the aspects of the cell wall microstructure and the exact location of polymers in muro in a variety of tissues [10]. The organization and structure of the cell wall, a determination of any differences in the composition of the cell walls that are related to cell development can be analyzed using antibodies [19].

Pectins are a heterogeneous group of polysaccharides [20] that are divided into several classes [14,21]. They contribute to the maintenance of the cell wall porosity, cell adhesion; have the ability to bind to ions, growth factors, and enzymes, and participate in cell differentiation and elongation [20,22]. The degree of esterification indicates the role of pectin in a cell wall function [23,24]. It has been proven that the degree of the methylation of pectins and their distribution is associated with the processes of cell division and cytodifferentiation [23,25]. Differences in the degree of the methyl esterification of homogalacturonan (HG) were observed during SE in *Chicorium* [26], *Musa* spp. [27], *Arabidopsis thaliana* [28], the *Cocos nucifera* callus [29], *Brachypodium distachyon* [30], and *Trifolium nigrescens* [31] embryogenic callus. Many studies that have been conducted on postembryonic plant growth have emphasized the role of the level of pectin esterification as a marker of the early stages of differentiation [25,32].

AGPs are primarily located in the outer surface of the cell membrane, in the cell wall, and in the intercellular spaces of various tissues and are actively secreted into a medium by suspension culture cells [33,34]. AGPs play an important role in modifying the spatial structure and chemical composition of the cell walls, which may be crucial in the process of cell differentiation [35]. Various patterns of the distribution of the AGPs epitopes have been investigated during the early stages of SE [36,37,38,39,40]. Some AGPs epitopes are involved in organogenesis in the androgenic callus of *Triticum aestivum* [41] or in *Centaurium erythraea* root culture [42], and have been postulated as being a good cytological marker that can be used to distinguish proembryogenic masses (PEM) from somatic embryos [43] and xylem differentiation [44,45].

It has postulated that extensins are involved in modifying the strength of the cell wall in the developmental and defensive contexts, and although they do not occur in large amounts, they can be a key component in the architecture of cell walls, particularly by increasing their strength [19]. It is believed that extensins also play a role during the plant developmental processes [45,46,47,48] and their adaptation to stress [49].

The process of SE in carrot has been intensively investigated. However, they have not as yet been analyzed intensively in the context of markers for cells that change the direction of differentiation. It has been shown that the AGPs epitopes that are recognized by the JIM4 and JIM8 antibodies bind to the cell surface of the pre-embryogenic masses of cells, which indicates that these epitopes are associated with the cells that switch the direction of their development from a somatic to embryogenic state [39,50]. Other studies led to the conclusion that the presence of these epitopes is not closely correlated with the embryogenic capacity of individual cells [51]. The importance of the contribution of the JIM8 epitope during carrot SE was clearly explained by McCabe et al. [52], who concluded that the epitope that is recognized by the JIM8 antibody can be used as a cytological marker for the very early stage of a cells transition into the embryogenic pathway. The presence and distribution of extensins during carrot SE has not yet been investigated (at least to the best knowledge of the authors), and, therefore, information about the involvement of these wall components during the induction phase of SE will provide new information.

During development and depending on the environmental conditions, the content of specific components of the cell wall changes, and, therefore, observations of the spatio-temporal modifications in the composition of cell walls can help to understand the mechanisms that control cell differentiation. SE is a convenient research model for analyzing the changes in cell fate and, thus, in the search for the wall markers that are associated with regaining totipotency, pluripotency, or callus formation (nomenclature according to Fehér [6]) is promising.

It has already been shown that some cell wall components can be markers of changes in cell fate, including the induction of SE [30] and the post-embryonal growth [32]. Immunohistochemical analysis of a *Brachypodium distahyon* callus culture demonstrated a decrease in the AGPs signal over the time of the culture as well as diverse extensin changes [30]. Moreover, it has been shown that the arabinogalactan protein (AGP) epitopes that are recognized by the JIM16 and LM2 antibodies, an extensin epitope that is recognized by the JIM11 antibody, and a pectic epitope that is recognized by the LM6 antibody, are positive markers for the embryogenic callus in *B. distahyon* [30]. It has also been revealed that the AGP epitope that is recognized by the MAC207 antibody is a good marker of morphogenic callus cells and that the epitope that is recognized by the LM2 antibody is a positive marker of the embryogenic cells in *F. tataricum* [53]. Studies during the SE of Arabidopsis revealed that the JIM8, JIM13, and JIM16 AGPs epitopes appeared to be the most specific for the cells reprogramming to the callus state and that the LM5 epitope is characteristic for cells present in the embryogenic domains [54]. It is postulated that the JIM4 epitope may be a marker for all of the stages accompanying the acquisition of embryogenic state by cells during carrot SE [39]. The non-methyl esterified (LM19) and methyl-esterified (LM20) HG together with LM5, LM6, JIM11, JIM12, JIM8, and JIM16 epitopes were described as characteristic for endosperm-derived cells that undergo dedifferentiation during the *Actinidia arguta* culture in vitro [55]. It has also been shown that the pectic epitope that is recognized by the LM8 antibody was absent, regardless of the morphogenic capacity of the *Actinidia arguta* callus [55].

Thus, the aim of the study was to investigate whether there is a relationship between the presence/absence of selected pectic, AGPs, and extensin epitopes and any changes in cell fate during the induction of SE in *Daucus carota* (L.) in order to answer the question of whether the analyzed wall constituents can be markers that can be used to track the developmental processes that accompany cell reprogramming. The uniqueness of the presented results is that it is a comprehensive spatio-temporal description of the changes in the wall composition in muro in cells undergoing reprogramming and change their fate.

## 2. Results

### 2.1. Explant Histology

At the beginning of the culture, typical tissues were detected in the hypocotyl explants: Epidermis, four-five layers of cortical cells, endodermis (which was not easy to distinguish on each section), pericycle (pericycle cells that were located on the xylem pole were large and elongated, resembling a pyramid shape), and stele (primary xylem and phloem; Figure S1A). After one day, no significant differences were observed compared to the beginning of the culture (Figure S1B). Between the 2nd and 5th day (Figure S1C–F), the pericycle cells that were located on the border between the phloem and xylem pole, next on the phloem and lastly on the xylem pole, began to divide. Before the first divisions, the cytoplasm increased in density (determined by the intensity of the coloration), the vacuole was traversed by numerous cytoplasmic strands, and the nucleus and nucleoli increased in size. The daughter cells from these divisions were characterized by dense cytoplasm (stained intensively with TBO), a large nucleus with a large nucleolus, and small vacuoles (Figure S1C). The pericycle cells that were located on the xylem pole divided anticlinally (Figure S1D). The endodermal cells also divided anticlinally (Figure S1E). Between the cells of the cortex, an increase in intercellular spaces was detected, which indicated the initiation of the process of their separation (Figure S1E–G). Between the 4th and 10th day of the culture, four clear poles with cells of a meristematic character (i.e., dense cytoplasm, a large nucleus with a large nucleolus, high cytoplasm-nucleus ratio; such features are postulated as characteristic for pluripotent cells, [56]) were visible (Figure S1E–G). After 10 days of the culture, the cells with meristematic character were visible around the entire circumference of the stele (Figure S1G). Numerous mitoses, which were oriented in different directions, were detected (Figure S1G). Within the meristematic part of the stele, cell complexes (easily distinguished by the thick cell wall surrounding them) were visible, which indicated that these cell resulted from the divisions of one mother cell (Figure S1E–G; insets). Within these complexes, cells with a large nucleus and nucleolus and numerous small vacuoles were present. After 10 days, only the stele remained compact, however, it had been modified structurally. One to two cell layers with a non-meristematic character were present on the surface (Figure S1G). The outer periclinal walls of the meristematic cells were thick and as a result, the border between them and surface cells was well visible (Figure S1G). On day 18 of the culture, a radial arrangement of the cells with a meristematic character was detected (Figure S1H). After 18 days of the culture (Figure S1H), several cell types could be distinguished: Separated cells with a round shape (Figure S1H; inset 1a); elongated, rod-shaped cells (Figure S1H; inset 1b) and other variously shaped cells (Figure S1H; inset 1c); surface cells with a non-meristematic character (Figure S1H; inset 2); cells that were dividing intensively with dense cytoplasm and several small vacuoles (Figure S1H; inset 3) and cells dividing intensively with dense cytoplasm and one large vacuole (Figure S1H; inset 4). The epidermis and cortex at that time point of the culture were completely separated and disintegrated.

### 2.2. Distribution of the Pectic Epitopes in the Explant

**Figure 1:**
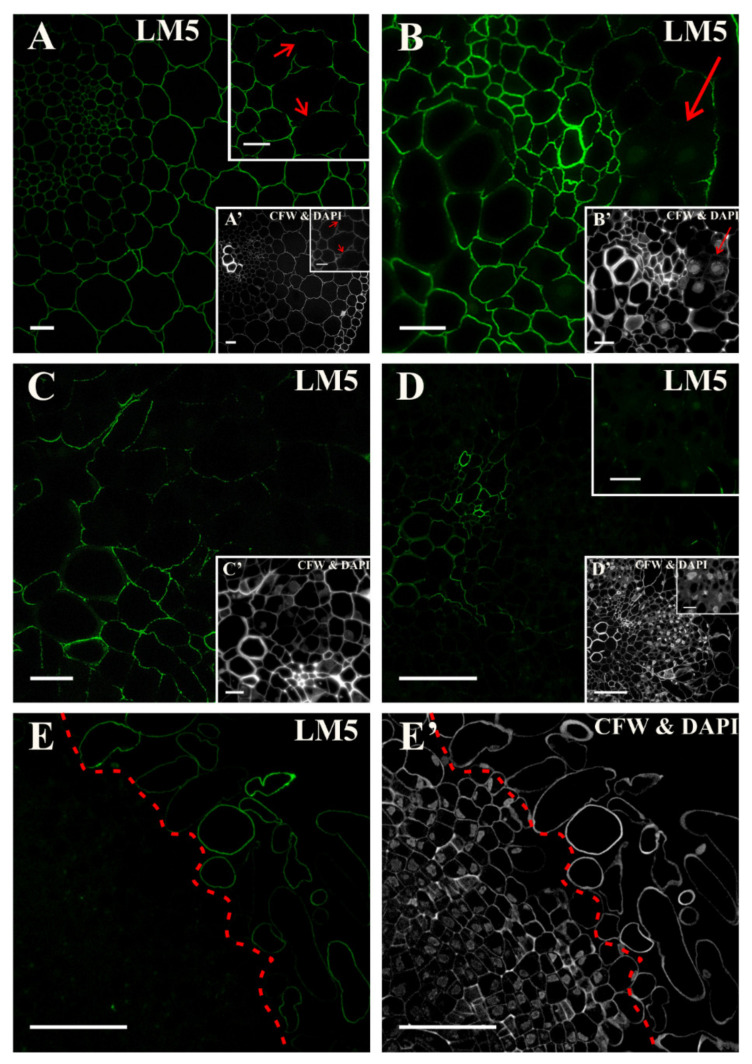
Distribution of the pectic epitope that is recognized by the LM5 antibody at different time points of the culture. (**A**) At the beginning of the culture continuous signal in the cell walls of all of the tissues except for the anticlinal walls of the pericycle cells that were located on the xylem side was detected (arrows, inset). (**B**) The hypocotyl after two days of culture with a clear signal in the cell walls of the vascular bundle, and almost lack of signal in the walls of the dividing cells (arrow). (**C**) After four days of the culture, the signal was detected in the cell walls of the vascular bundle; in the walls of the cells that had divided, the signal was almost not detected. (**D**) After five days of the culture, the signal was not detected in the walls of the dividing cells (inset). (**E**) On day 18, a clear signal characterized the walls of the separated cells. The dashed line separates the location of the epitope. (**A’**–**E’**) Calcofluor white (CFW) and DAPI staining of the section shown on **A**–**E**. Scale bars: **A**, **A’**—20 μm; inset—10 μm; **B**, **B’**; **C**, **C’**—10 μm; **D**, **D’**—50 μm; inset—10 μm; **E**, **E’**—50 μm.

In the explant, prior to its transfer to the induction medium, the epitope that was detected by the LM5 antibody was located in the walls of all of the cell types except the anticlinal walls of pericycle cells that were located on the xylem side (Figure 1A, inset, Figure S2A). After the first day of the culture, a continuous, fluorescent signal was present in all of the cells (not shown). On the second day of the culture, the fluorescent signal in the inner walls of the cell clusters, which were the derivatives of the dividing pericycle cells, was much less pronounced (Figure 1B and inset). Staining with calcofluor and 4′,6-diamidino-2-phenylindole (DAPI) indicated that the loss of the signal that was generated by the LM5 antibody occurred in the cells with a meristematic character (Figure 1B inset). From days four and five of the culture, complete disappearance of the epitope recognized by the LM5 antibody was detected in the walls of most of the dividing cells, and its presence was primarily limited to the walls of the vascular tissue (Figure 1C,D and insets). After 18 days, the signal was present only in the detached cells ((Figure 1E; Figure S2A, Figure S3A); Table S1).

**Figure 2:**
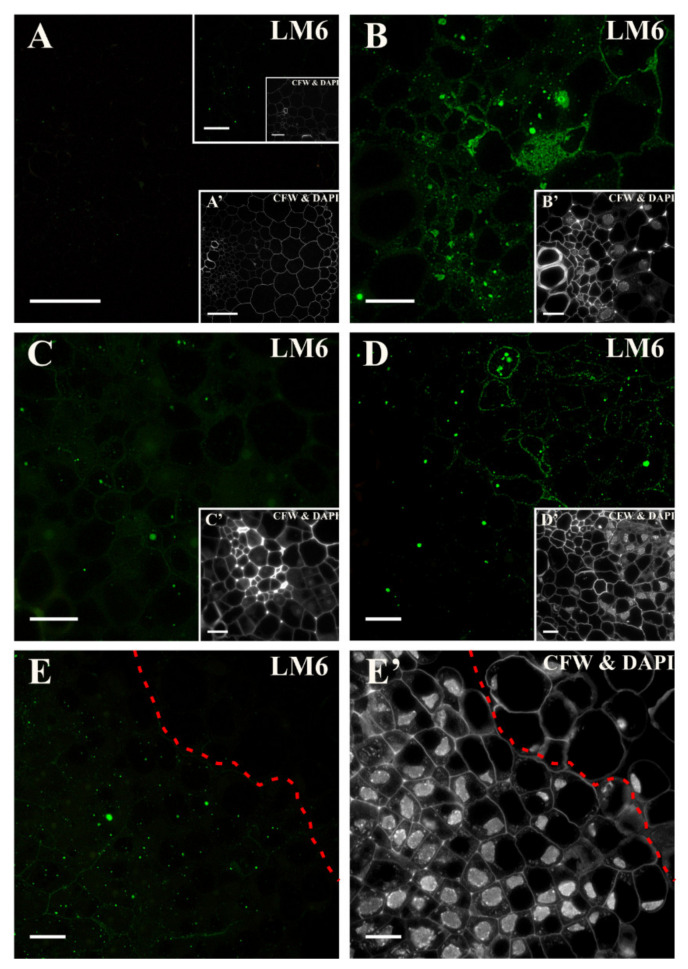
Distribution of the pectic epitope that is recognized by the LM6 antibody. (**A**) At the beginning of the culture, only a few punctate signals in the walls and cytoplasm in the cells of various tissues were detected. (**B**) After two days of culture, clear punctate signals in the cell walls and cytoplasmic compartments of the dividing cells and the vascular bundle were present. (**C**) On the fourth day of culture, the single punctate signals were detected in the dividing cells. (**D**) Presence of the signal on the fifth day in walls and in the cytoplasmic compartments of the cells with dense cytoplasm. (**E**) At the end of the culture (18 days), a punctate signal in the cytoplasmic compartments of the dividing cells in the surface layers of the stele was detected. The dashed line separates the occurrence of the epitope. (**A’**–**E’**) Calcofluor white (CFW) and DAPI staining of the section shown on **A**–**E**. Scale bars: **A**, **A’**—50 μm; inset—10 μm; **B**–**E**, **B’**–**E’**—10 μm.

At the beginning of the culture, the epitope that is detected by the LM6 antibody was not present except for sparse punctate signals in the stele (Figure 2A and insets, Figure S2B, Figure S3B). After two days, a signal was found in the walls and in the cytoplasm compartments of the dividing pericycle cells (Figure 2B). After four days, the punctate signal was only detected in the cytoplasm compartments of some of the cells, indicating the disappearance of this epitope during the culture (Figure 2C and inset). After five days, a clear punctate signal was detected in the dividing cells with dense cytoplasm, while in highly vacuolated cells, the epitope was much less abundant (Figure 2D and inset). On the 18th day of the culture, the signal was primarily detected in the cytoplasmic compartments of the cells that were dividing intensively, while no signal was detected in the separated cells (Figure 2E,E’, Figure S2B, Figure S3B; Table S1). To summarize, the pectic epitope that was recognized by the LM6 antibody was not a constitutive component of the walls of the carrot hypocotyl cells of any of the tissues. Under culture conditions, this epitope was synthesized (as was first detected in the cytoplasmic compartments), and afterward, it was present in the walls of the dividing cells in the form of a punctate signal. Thus, this epitope could be a positive marker of cells that regain their meristematic/pluripotent potency and a negative marker of detached cells that were not involved in SE.

**Figure 3:**
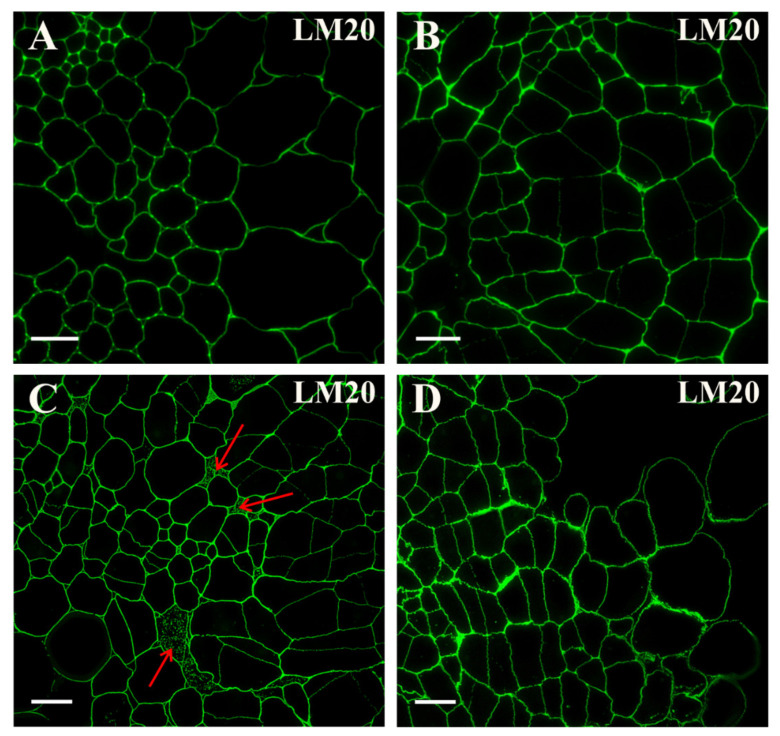
Distribution of the pectic epitope that is detected by the LM20 antibody in the explant cells. (**A**) Signal presence in the cell walls of all of the tissues. (**B**) After four days of the culture signal still present in the walls of all of the cells. (**C**) On the next day, the continuous wall signal in all of the explant cells and punctate signals in intercellular spaces (red arrows) were detected. (**D**) The hypocotyl after 18 days of the culture with clear continuous wall signal in all of the explant cells, including the separated ones. Scale bars: (**A**–**D**)—10 μm.

From the start of the culture, a clear fluorescence signal indicating the presence of the epitope that is recognized by the LM20 antibody was detected in all of the explant cell walls (Figure 3A, Figure S2C, Figure S3C). This signal persisted throughout the culture on the induction medium. The only noticeable difference was a less pronounced signal in the newly formed walls of the dividing cells (resulting from the thinness of the new walls; Figure 3B). A clear signal was detected in intercellular spaces (Figure 3C), in the explant surface cells and separated cells (Figure 3D; Table S1).

**Figure 4:**
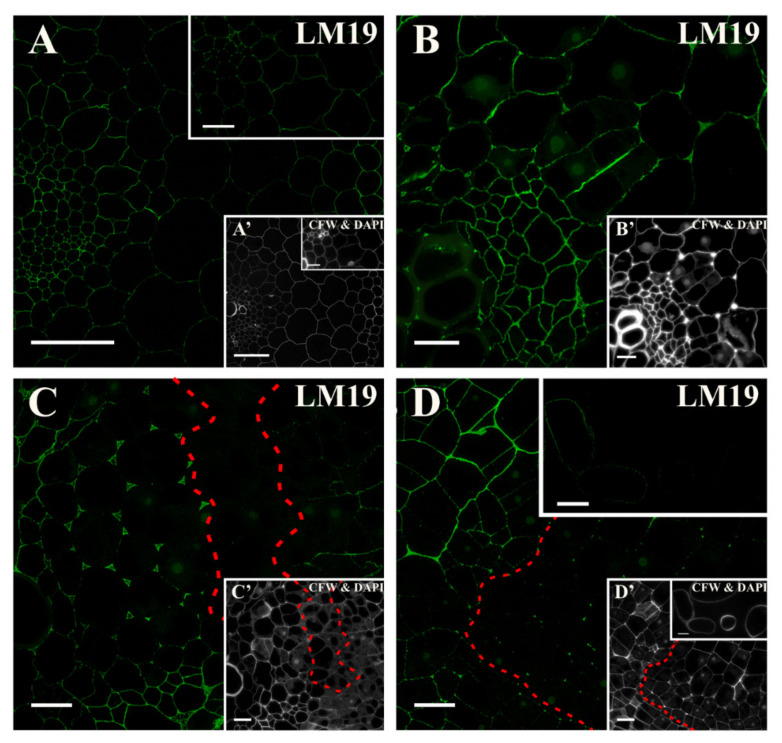
Distribution of the pectic epitope that is detected by the LM19 antibody at different time points of the culture. (**A**) The hypocotyl at the start of the culture with noticeable signal in the cell walls of all of the tissues (inset-magnification of a stele). (**B**) After two days of the culture signal in the newly formed walls was not detected but the epitope starts to be present in the developing intercellular spaces. (**C**) From the fifth day of culture, the presence of the signal in the intercellular spaces was abundant, but in dividing cells was not detected (area separated by a dashed line). (**D**) At the end of the culture, the signal was present only in dividing cells with a large vacuole (area separated by a dashed line) and in the walls of some of the separated cells (inset). (**A’**–**D’**) Calcofluor white (CFW) and DAPI staining of the section shown on **A**–**D**. Scale bars: **A**, **A’**—50 μm, inset—10 μm; **B**–**D**, inset, **B’**–**D’**—10 μm.

A low-methyl-esterified HG epitope that is recognized by the LM19 antibody was present in the cell walls of all of the tissues from the beginning of the culture (Figure 4A and inset, Figure S2D, Figure S3D). After the first two days of the induction, the epitope began to occur in the intercellular spaces (Figure 4B). In the following days of culture, in the walls of dividing cell, signal was not detected (Figure 4C). The association of the analyzed epitope with the formation of the intercellular spaces was observed (Figure 4C and inset). The LM19 epitope was located in the emerging intercellular spaces (Figure 4C and inset). After 10 days of the culture, the signal only remained in some of the dividing cells without a meristematic/pluripotent character (Figure 4C,D). The signal was also present in some of the separated cells (Figure 4D and inset; Table S1).

**Figure 5:**
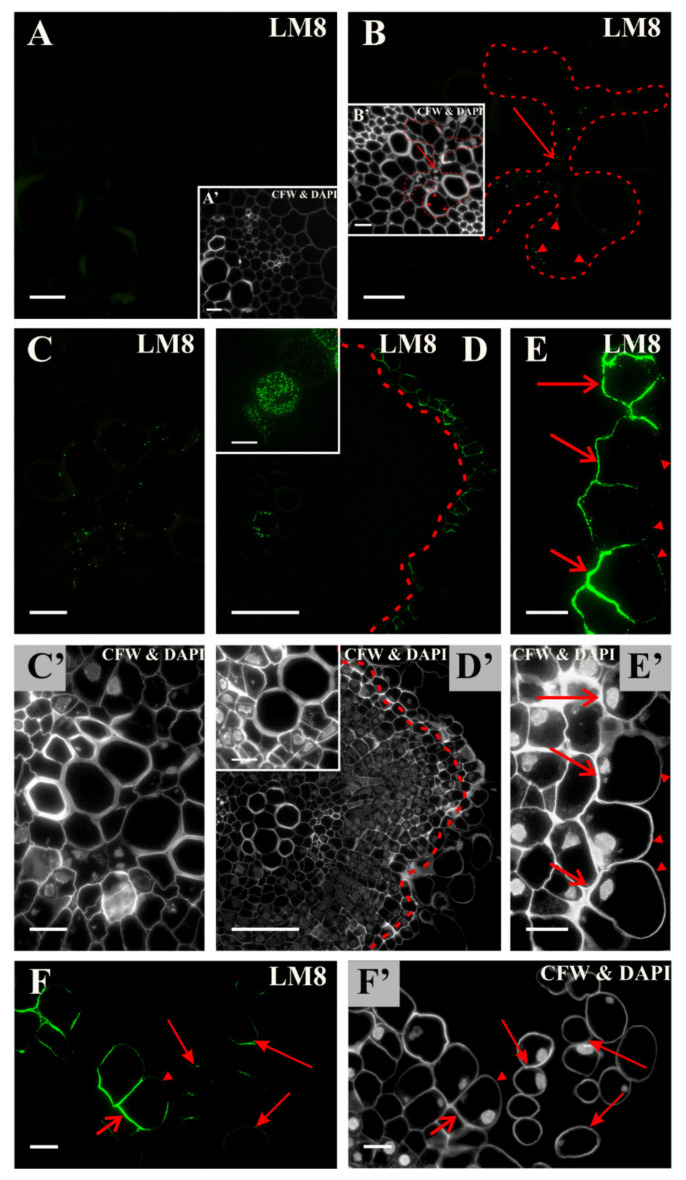
Distribution of the LM8 pectic epitope during the culture. (**A**) At the start of the culture, this epitope was not present in any of the explant cells. (**B**) After two days, a punctate signal in the cytoplasmic compartments of the xylem parenchyma (arrow) and in the walls of mature vessels (arrowheads; the area of the occurrence of the epitope is marked by a dashed line) was detected. (**C**) Presence of the signal (the fourth day) in the walls of the vessels. (**D**) On the tenth day, a noticeable continuous signal in the walls of the cells separating (area marked with a dashed line) and inside the differentiating vessels was detected (inset). (**E**,**F**) Presence of the signal in the walls of the separated cells (arrows) and in the part of the walls from the surface (arrowheads) after 18 days. In the separated cells, the signal was only detected in some parts of the walls (full arrows). (**A’**–**F’**) Calcofluor white (CFW) and DAPI staining of the section shown on A-F. Scale bars: **A**–**C**, **A’**–**C’**, **D**, **D’** inset; **E**,**F**, **E’**,**F’**—10 μm; **D**, **D’**—50 μm.

The HG epitope that is recognized by the LM8 antibody was not present in any of the explant cells at the start of the culture (Figure 5A and inset, Figure S2E, Figure S3E). During the culture, the signal appeared in the cytoplasmic compartments in the xylem parenchyma and differentiating vessel elements (Figure 5B,D and insets, Figure 5C,C’). After 10 days, there was a noticeable signal in the anticlinal and inner periclinal walls of the cells that were predestined to detach, while in the outer periclinal walls, the signal was almost not present (compare Figure 5D,E,F with D’,E’,F’). In a few of the separated cells, this epitope was located only in parts of cell walls (Figure 5F,F’; Table S1). This epitope is not a marker of cells reprogramming to meristematic/pluripotent state, but is a positive marker of the cells that are undergoing separation from the explant, thus not involved in SE.

### 2.3. Distribution of the AGP Epitopes

**Figure 6:**
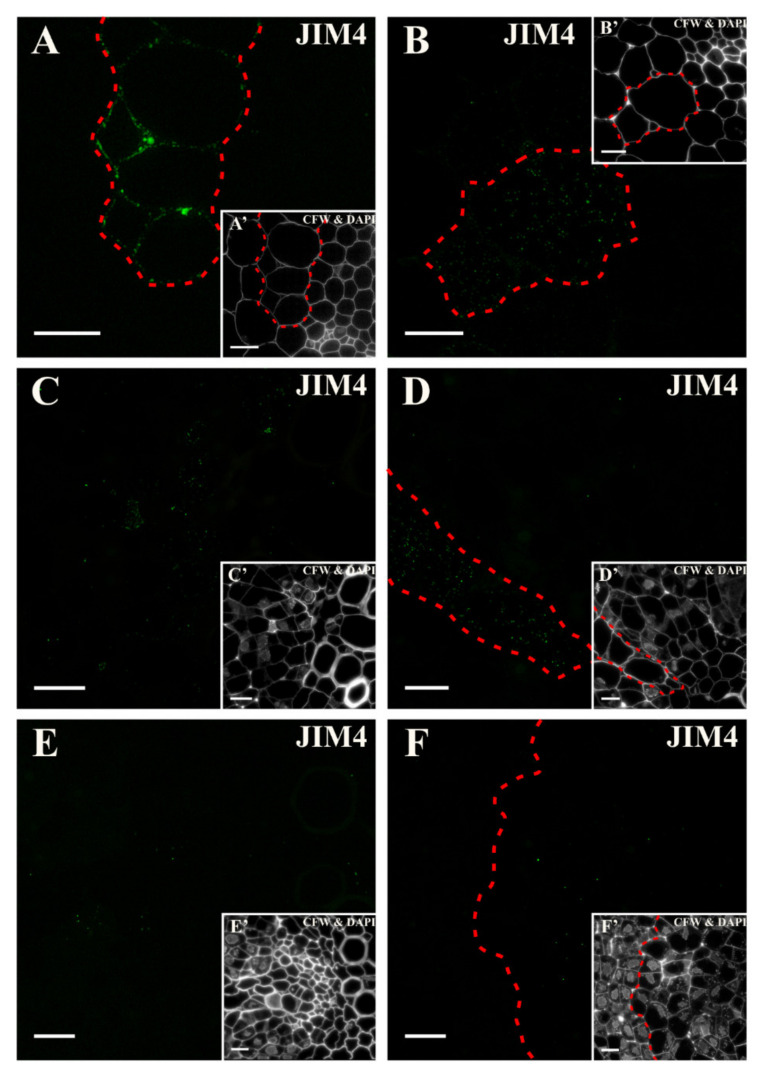
Distribution of the arabinogalactan protein (AGP) epitope that is detected by the JIM4 antibody at different time points of the culture. (**A**) At the beginning, the signal was present in the pericycle cell walls that were located on the xylem side (area marked with dashed line). (**B**) After two days, only a punctate signal was detected in the cytoplasmic compartments of the pericycle cells (area marked with dashed line). (**C**) From the fourth day, a punctate signal in the cytoplasmic compartments of some explant cells was detected. (**D**) On the next day, a punctate signal inside some of the highly vacuolated cells was present (area marked with dashed line). (**E**,**F**) Signal disappearance in the following days of the culture was noticed (**E**—10 days; **F**—18 days). (**A’**–**F’**) Calcofluor white (CFW) and DAPI staining of the section shown on **A**–**F**. Scale bars: **A**–**F**, **A’**–**F’**—10 μm.

Before transferring the explant to the induction medium, the epitope that was detected by the antibody JIM4 occurred only in the walls of the pericycle cells that were located at the xylem pole of the vascular bundle (Figure 6A and inset, Figure S2F, Figure S3F). After two days, a punctate signal was present in the cytoplasmic compartments of some of the pericycle cells (located at the xylem pole) that had not yet been divided (Figure 6B and inset). From the following days (4th and 5th), the signal was punctate and localized in the cytoplasmic compartments of the highly vacuolated cells (Figure 6C,D and insets). The signal disappeared with the extension of the culture duration (Figure 6E and inset). After day 18, the epitope was hard to detect in the explant cells (Figure 6F and inset; Table S1).

**Figure 7:**
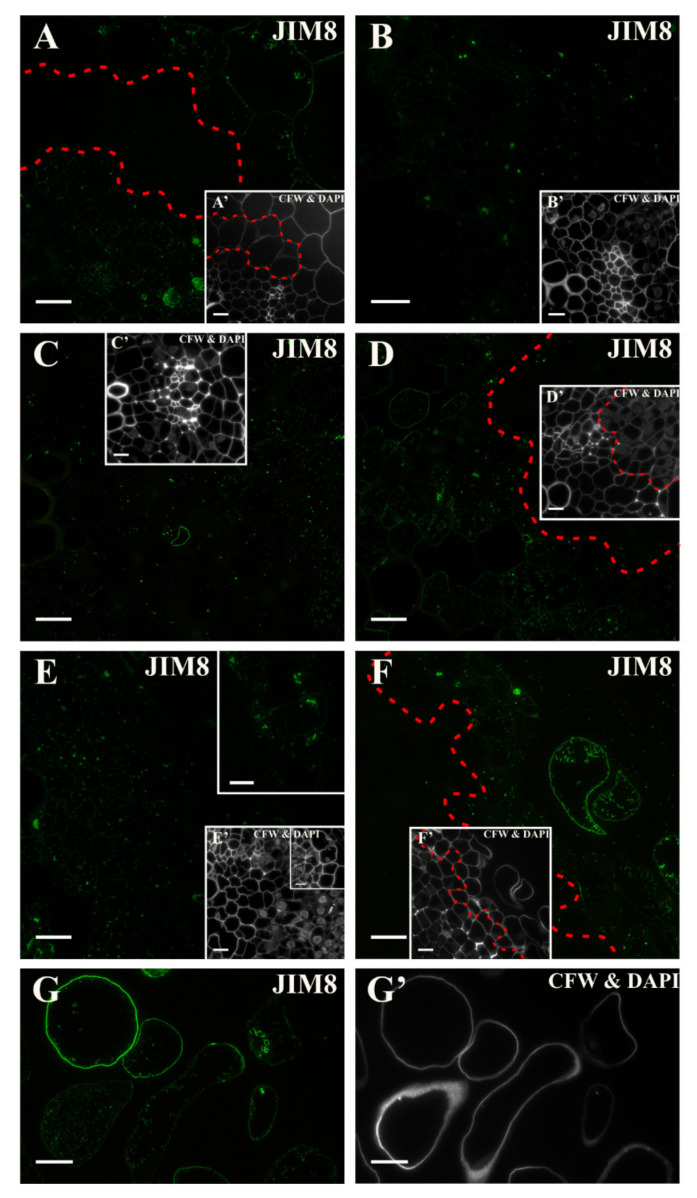
Distribution of the AGP epitope that is recognized by JIM8 antibody at different time points of the culture. (**A**) A punctate signal was detected in the cell walls of some cells and cytoplasmic compartments of all of the explant cells except for the pericycle cells that were located on the xylem side (area marked with dashed line). (**B**,**C**) Presence of a punctate signal in the cytoplasmic compartments (**B**—3 days, **C**—4 days of the culture). (**D**) After five days, the occurrence of a signal in the phloem and parenchyma cells was detected. In the dividing cells with a large nucleus (area separated by dashed line), the signal was almost not present. (**E**) After 10 days of the culture, a punctate signal was detected mainly in the parenchyma cells and in the walls and the cytoplasmic compartments of the separated cells (inset). No signal in the cells with meristematic features was found. (**F**) At the end of culture a signal in the cytoplasmic compartments of the cells that are located at the surface of the explant and also in the separated cells was detected (area separated by a dashed line). (**G**) Higher magnification of the separated cells with signal present in the walls and in the cytoplasmic compartments of detached cells. (**A’**–**G’**) Calcofluor white (CFW) and DAPI staining of the section shown on **A**–**G**. Scale bars: **A**–**G**, **A’**–**G’**—10 μm.

At the start of the culture, the AGP epitope that was recognized by the JIM8 antibody was distributed in all of the explant cells except for the pericycle cells that were located on the xylem pole of the vascular bundle (Figure 7A and inset, Figure S2G, Figure S3G). In the other cells, the signal was present in both the cell walls and in the cytoplasmic compartments (Figure 7A). After about three days of culture, a punctate signal was detected in the phloem and dividing pericycle cells (Figure 7B and inset). During the following days of the culture, single punctate signals were observed in the dividing cells as well as in the phloem and parenchyma (Figure 7C,D,E, and insets). At the end of the culture, the signal was present in both the cell wall and in the cytoplasmic compartments in the separated cells but was not detected in the dividing cells with meristematic features (Figure 7F and inset; G,G’; Table S1). Thus, it can be stated that this epitope is a negative marker of meristematic/pluripotent cells and a positive marker of separated cells.

**Figure 8:**
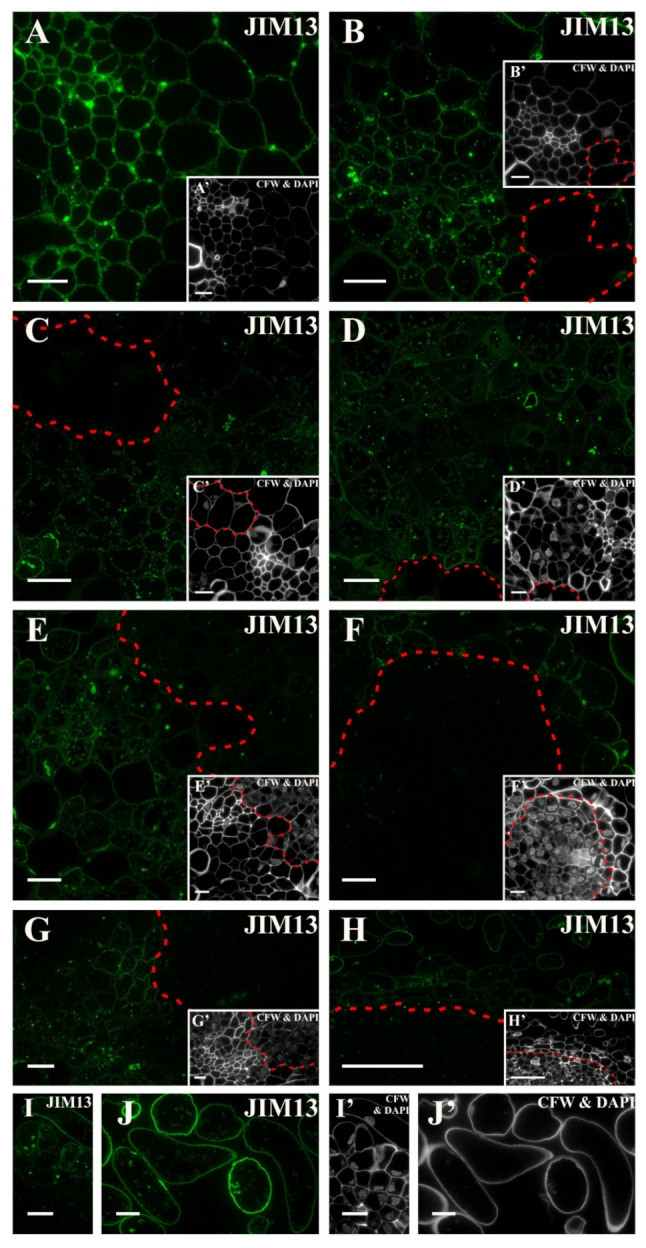
Distribution of the AGP epitope that is detected by the JIM13 antibody. (**A**) At the beginning of the culture, a clear signal in the cell walls of all of the explant tissues was observed. (**B**) After one day of the culture, signal was detected in the cell walls as well as in the cytoplasmic compartments of all of the cells except the pericycle cells that were located on the xylem pole (area marked with a dashed line). (**C**) On the second day, a similar epitope distribution was detected as in an earlier stage. No signal was found in the elongated pericycle cells (area marked with a dashed line). (**D**) After three days, the signal was abundantly present in the cell walls and in the cytoplasmic compartments of the dividing cells (area marked with a dashed line). (**E**) From the fifth day, the occurrence of a signal in the cell walls, cytoplasmic compartments of the phloem elements, and parenchyma, but signal disappearance in the dividing cells with meristematic features was noted (area separated by a dashed line). (**F**) The hypocotyl after 10 days of the culture with the pronounced signal in the separated cells and progressive loss of signal in the dividing cells (the dashed line separates both areas). (**G**) After 18 days, the loss of signal in meristematic cells was noticed (area separated by a dashed line). (**H**) Presence of signal in the walls and cytoplasmic compartments of the cells that were predestined to detach and the cells that had separated from the explant. In the meristematic cells, the signal was not found (area separated by a dashed line). (**I**) Higher magnification of the cells that were predestined to detach. (**J**) Higher magnification of the separated cells. (**A’**–**J’**) Calcofluor white (CFW) and DAPI staining of the section shown on **A**–**J**. Scale bars: **A**–**G**, **A’**–**G’**,**I**, **J**, **I’**, **J’**—10 μm; **H**, **H’**—50 μm.

The fluorescence signal indicating the presence of the epitope that is detected by the JIM13 antibody was present in the cell walls of all of the hypocotyl tissues in the explant at the beginning of the culture that indicates that this epitope is a constitutive wall component for the carrot hypocotyl (Figure 8A and inset, Figure S2H, Figure S3H). After the first day on the induction medium, the epitope was not found in the pericycle cells on the xylem pole. (Figure 8B and inset). After two days of culture, the signal was present in both the cell walls and in the cytoplasmic compartments in many of the cortex cells but was not detected in the pericycle cells undergoing elongation (Figure 8C and inset). A similar distribution of this epitope was found after three days (Figure 8D and inset). As the culture lengthened, the signal disappeared in cells that re-gained the meristematic/pluripotent character (Figure 8E, G, and insets). In the following days, the presence of the epitope was detected in the walls of the cells that were predestined to detach (Figure 8F and inset) and in the phloem elements as well as in the adjacent dividing cells (Figure 8G and inset). At the end of the culture in intensively dividing cells signal disappeared, whereas the signal was well visible in the walls and cytoplasmic compartments of cells that were predestined to detach and the cells that had separated from explant (compare Figure 8G and inset, I,J with I’,J’; Table S1).

**Figure 9:**
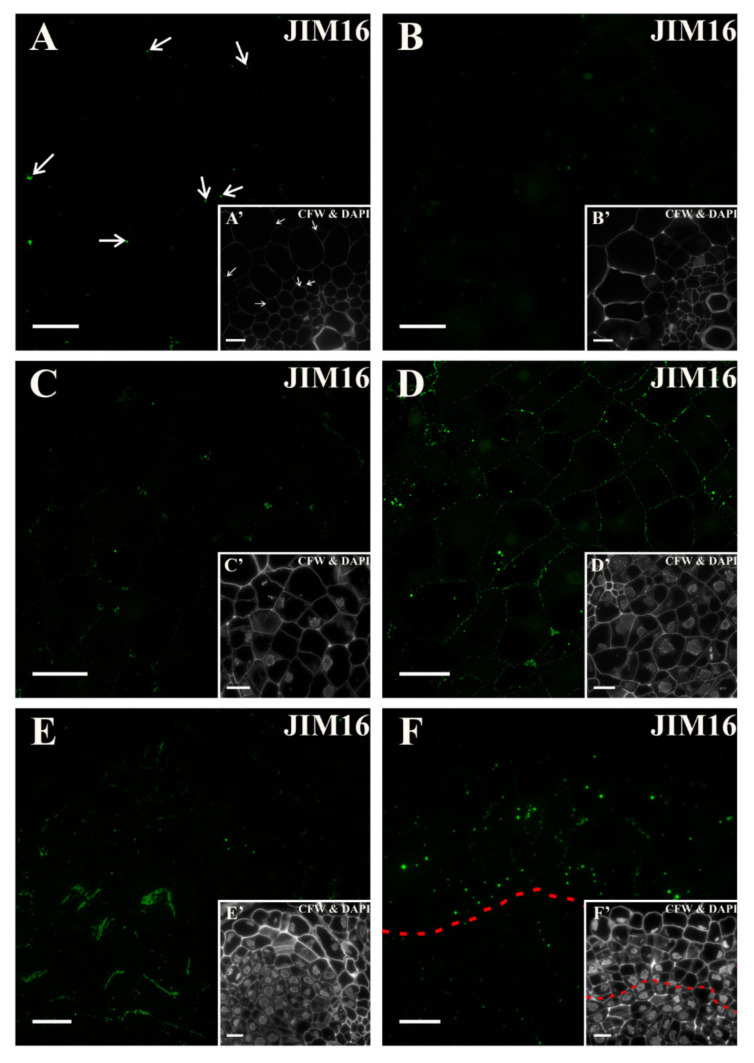
Distribution of the AGP epitope that is detected by the JIM16 antibody. (**A**) At the beginning of the culture, the signal was absent, except for a few punctate signals in some cells (arrows). (**B**,**C**) During the next days, the punctate signal was rarely observed (**B**-second day of the culture; **C**-after four days). (**D**) After five days of the culture, signal was detected mainly in the walls of the dividing cells. (**E**) Along with increasing the duration of the culture, the signal disappearance in the dividing cells was noticed. (**F**) At the end of the culture, the punctate signal was detected mostly in the cells of the surface layers of explant (area separated by a dashed line). (**E’**,**F’**) Calcofluor white (CFW) and DAPI staining of the section shown on **E**, **F**. Scale bars: **A**–**F**, **E’**, **F’**—10 μm.

At the beginning of the culture, the epitope that was recognized by the JIM16 antibody was not detected (Figure 9A, Figure S2I, Figure S3I). Sparse, punctate signals were present in both the wall and the cytoplasmic compartments of the cortex cells (Figure 9B,C). With the increasing number of pericycle cell divisions, the signal was detected in the walls of dividing cells (Figure 9D). The signal was also present in the cells that were detaching (Figure 9E and inset). After 18 days on the induction medium, the signal was detected in a few surface cell layers, and at that time, the loss of signal in walls of meristematic cells was noticeable (Figure 9F and inset; Table S1). Such results may indicate that this epitope was not constitutive of the carrot hypocotyl, and that was a positive marker of dividing cells.

**Figure 10:**
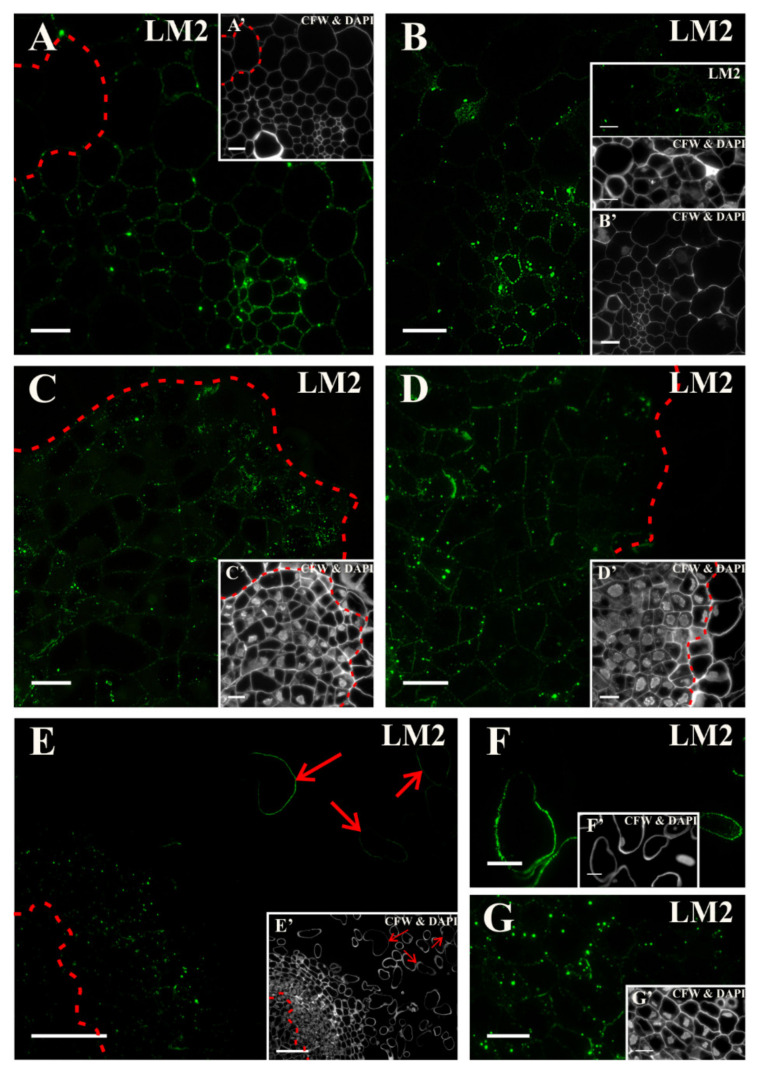
Distribution of the AGP epitope that is detected by the LM2 antibody. (**A**) At the start of the culture, a well visible signal was detected in the cell walls of all of the tissues except for the pericycle cells that were located on the xylem pole (area marked with a dashed line). (**B**) The presence of a signal in the walls and cytoplasmic compartments of the dividing pericycle cells (also inset) as well as in the phloem and xylem elements after two days of culture was noticed. (**C**) The signal distribution in the walls and vacuoles of the dividing cells after five days (area marked with a dashed line). (**D**) A pronounced signal in the cell walls, cytoplasmic compartments, and vacuoles of the dividing cells was detected after 10 days of the culture. In the surface layers, the cells were devoid of signal (areas separated by a dashed line). (**E**) At the end of the culture, the signal was present in cells in a few surface layers (area separated by a dashed line) and in cells that were separating (arrows). (**F**) Higher magnification of separated cells. (**G**) Higher magnification of cells that were predestined to separate. (**A’**, **B** inset), (**C’**–**G’**) Calcofluor white (CFW) and DAPI staining of the section shown on **A**, **B** inset, Scale bars: **A**–**D**, **A’**, **C’**, **D’**, **G**, **F**, **G’**, **F’**—10 μm; **E**, **E’**—50 μm.

Before transferring the explant to the induction medium, a signal that was generated by the LM2 antibody was detected in the walls of all of the explant cells except for the elongated pericycle cells (Figure 10A, Figure S2J, Figure S3J). After transferring the explant into the induction medium, the signal started to be also seen in the dividing pericycle cells (Figure 10B). In the following days, this epitope was mainly detected in the walls and cytoplasmic compartments of the intensively dividing cells, but it was missing in the cells that were predestined to detach (Figure 10C,D). After 18 days of the culture, a punctate signal was observed in cytoplasmic compartments of the surface cells that were predestined to detach as well as in some of the separated cells (Figure 10E,F,G, and insets; Table S1). Thus, it can be concluded that this epitope is a: Negative marker of pericycle cells from the xylem pole, a positive marker of cells that re-gained the pluripotency (at least up to the tenth day of the culture), and cells undergoing separation. Moreover, it can be stated that this epitope was a negative marker of competent cells, however, it must be confirmed on the genetic level.

### 2.4. Extensins

**Figure 11:**
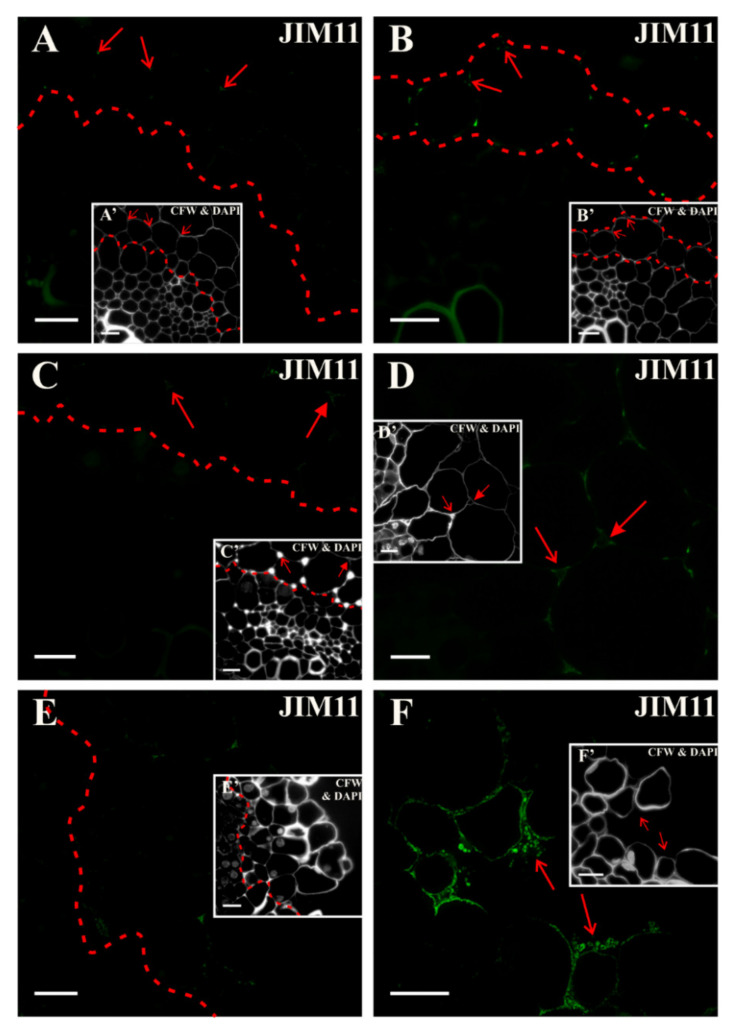
Distribution of the extensin epitope that is detected by the JIM11 antibody. (**A**) At the beginning of the culture, the signal was detected only at the junction of the walls between the cells that are located outside the pericycle (arrows; the dashed line separates the area of the occurrence of the epitope). (**B**,**C**) In the following days of the culture (**B**—two days, **C**—four days), the signal was distributed in a similar pattern as in previous days (areas marked with dashed lines, arrows point to intercellular spaces). (**D**) After five days of the culture, the signal was detected in the emerging intercellular spaces (full arrow) and at the junction of the cell walls (arrow). (**E**) From the tenth day of the culture, the signal was almost not detected, except for cells predestined to detach (area separated by a dashed line). (**F**) Separated cells after 18 days with a clear signal in the cell walls and outside the cells (arrows). The green color of the vessel walls is an autofluorescence. (**A’**–**F’**) Calcofluor white (CFW) and DAPI staining of the section shown on **A**–**F**. Scale bars: **A**–**F**, **A’**–**F’**—10 μm.

The extensin epitope that was recognized by the JIM11 antibody at the beginning of the culture appeared to very limited places and was located in the cell corners between the anticlinal and periclinal walls in the cortex layer of cells adjacent to the pericycle (Figure 11A and inset). A similar distribution of this epitope was observed after two days of the culture (Figure 11B and inset, Figure S2K, S3K). After the subsequent days, an epitope was observed in the emerging intercellular spaces between cells predestined to detachment (Figure 11C and inset). On the 10th day of the culture, the signal disappeared in the explant (Figure 11E and inset; Table S1). At the end of the culture, a clear signal was observed in the walls of detached cells and outside the cell walls (Figure 11F and inset). It can be concluded that this epitope was not a constitutive component of the cell walls but was a positive marker of the cell separations process. Since this epitope was also located outside the cell walls, which indicated its secretion into the medium, it may suggest that it participated in the transduction of some signals regulating cell differentiation, but this statement required experiments, probably with the use of cell wall mutants.

**Figure 12:**
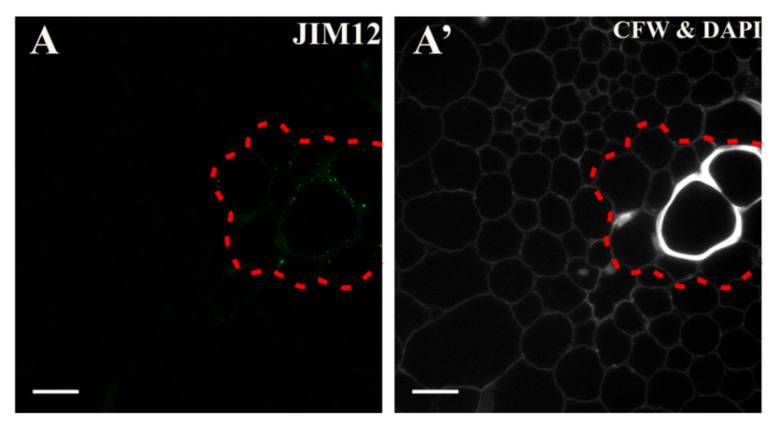
Distribution of the extensin epitope that is detected by the JIM12 antibody (**A**) Fragment of the hypocotyl vascular bundle. A punctate signal in the cell wall of the xylem elements was visible (area marked with a dashed line). (**A’**) Calcofluor white (CFW) and DAPI staining of the section shown on A. Scale bars: **A**, **A’**—10 μm.

In the explant, the epitope that is detected by the JIM12 antibody was present only in the vessels and differentiating vessel elements independent of the culture duration (Figure 12A; Figure S2L, Figure S3L, Table S1).

**Figure 13:**
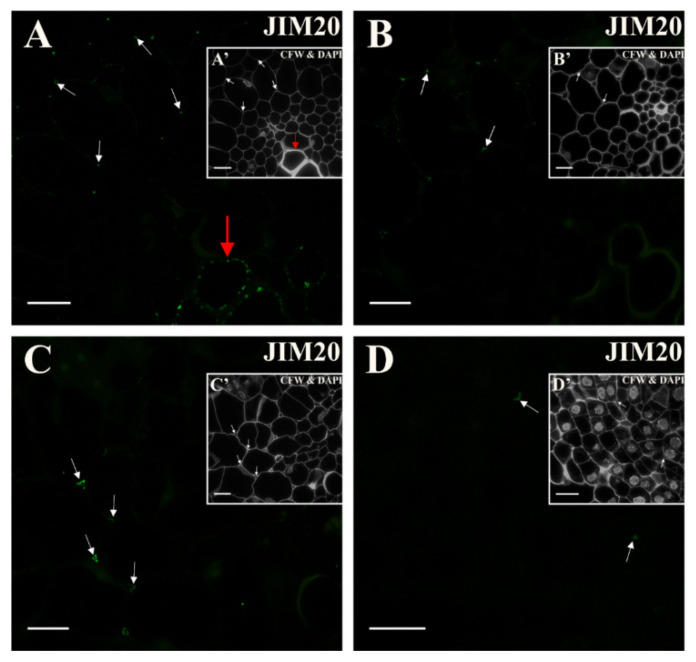
Distribution of the extensin epitope that is detected by the JIM20 antibody in the explant cells (**A**) At the start of the culture signal was detected in the cell corners between the pericycle cells (white arrows) and in the walls of the vessels (red arrow). (**B**) After two days signal was present only in some of the intercellular spaces between the elongated pericycle cells (arrows). (**C**) In the following days signal was detected only in some of the intercellular spaces between the cells that were undergoing their first division (arrows). (**D**) From the 10th day of the culture signal disappeared (arrows). Scale bars: **A**–**D**—10 μm.

After immunostaining with the JIM20 antibody, a fluorescence signal was barely visible and present in a few places of the explant, especially in the cell corners (Figure 13A, Figure S2M, Figure S3M). In the following days of the culture, the signal disappeared in the walls of the vessels (Figure 13B). The most pronounced presence of the epitope was found on the fifth day of the culture in the emerging intercellular spaces (Figure 13C). The signal disappeared from the 10th day of culture (Figure 13D; Table S1; after 18 days, no fluorescence signal was detected in any of the cells types—not shown).

### 2.5. Results Summary

The pectic epitope that was recognized by the LM5 antibody was a positive marker of the detached cells and a negative marker of the meristematic cells and formative divisions. Thus, this epitope can be used to detect the cells that were reprogramming their state from somatic to meristematic/pluripotent in explants and as a marker of the cells that are not involved in SE (Table 1, Table S2; performed quantitative analysis confirmed the identified epitopes as markers of various cellular events). The LM6 epitope was a constitutive component of pectin in the cell walls of carrot hypocotyl, a positive marker of the formative divisions and the cells that regained a meristematic state and negative marker of cells undergoing separation and detached cells (Table 1, Table S2). The LM20 epitope was a constitutive component of pectin in the cell walls of the carrot hypocotyl (Table 1, Table S2). The LM19 epitope was a constitutive component of cells and no spatio-temporal changes were observed in its distribution during the culture (Table 1, Table S2). The epitope that was recognized by the LM8 antibody was a positive marker of the cells that were separating from the explant (Table 1, Table S2). The JIM4 epitope was not a constitutive component of the carrot hypocotyl independent of the culture duration but was a positive position marker of the pericycle cells from the xylem pole (Table 1, Table S2). The JIM8 antibody was a positive marker of the cells predestined to separation and separated cells but a negative cell position marker of the pericycle cells that were located on the xylem pole and cells reprogramming to the meristematic/pluripotent state (Table 1, Table S2). Moreover, this epitope was not a constitutive wall chemical component. The AGP epitope that was recognized by the JIM13 antibody was a constitutive component of carrot hypocotyl cell walls and was a positive marker of cells that were separating as well as formative divisions, but was a negative marker of the cells that were reprogrammed into the meristematic state (Table 1, Table S2). The JIM16 epitope was not a constitutive wall component and can be a positive marker of formative divisions (Table 1, Table S2). The LM2 epitope was a positive marker of the cells that had regained the meristematic state, the formative divisions, and separating cells but a negative marker of the cell position of the pericycle cells on the xylem pole (Table 1; Table S2). The JIM11 antibody was a positive marker of the cells that were detaching and may also be involved in cell signaling (Table 1, Table S2). The JIM12 and JIM20 epitopes were not constitutive components of the explant cells and were not involved in any developmental process taking place during the culture (Table 1, Table S2).

A positive marker indicates that it was detected or appeared de novo during the culture for a given epitope, while a negative marker indicates that a given epitope was not present or lost during culture.

## 3. Discussion

### 3.1. Morpho-Histological Changes in an Explant that Had Been Subjected to SE Induction

The process of SE is intensively studied because it can be used to produce plants in vitro and to analyze the mechanisms that regulate the process of somatic embryo formation in order to explain the developmental changes during zygotic embryogenesis. One other aspect of SE is also important. Namely, during SE, there are changes in the direction of cell differentiation. Meristematic, embryogenic, and callus cells are formed from explant cells. During SE, cytodifferentiation in a new direction and pattern formation took place. Cells that do not change their fate are also present and can be used to search for markers to indicate cells that are not competent. Therefore, SE provides an opportunity to explore the mechanisms that underlie cell differentiation in connection with their reprogramming. Because explant cells are not meristematic/embryogenic per se, the induction phase is required for cells to regain their meristematic/pluripotent and embryogenic/totipotent character [57]. As this phase is necessary to initiate changes in the explant and force cell differentiation in a new direction, therefore, this stage was analyzed in this study.

Many mechanisms that regulate changes in the direction of cell differentiation, e.g., epigenetic control [58,59,60], the influence of hormones [61,62], or symplasmic communication between cells that are performing various developmental programs [63] are indicated as being involved in regulating the changes in cell fate. Knowledge about the cytological markers, including the markers in the cell walls, will be helpful for the study of molecular mechanisms regulating the cell fate transition (for review see Fehér et al. [64]).

The role of the chemical and structural reorganization in the cell walls in the context of the control and/or as markers of changes in the direction of cell differentiation is relatively unknown. It is particularly important to look for markers enabling phenotype identification (including wall markers) of cells that implement various development programs. This knowledge is particularly important for recognizing cells that change the direction of their differentiation, which will facilitate the analysis of cells at the molecular and/or genetic level.

Research attempting to understand the changes in the cell walls that accompany cell differentiation is extensive, but results that are presented here indicate those components of the cell wall that can be used to analyze the early stages of cell fate reprogramming on a molecular level. Moreover, in the conducted analyses, a large set of antibodies was used to detect the various types of pectins, AGPs and extensins, which provides a broader picture of the developmental changes in the chemical composition of the cell walls during cell reprogramming.

### 3.2. Cytological Identification of the Explant Cells That are Undergoing Reprogramming

During the carrot hypocotyl culture, the epidermal cells and, subsequently, the cortex cells of the explant were separated. Studies performed on the same type of explant have shown similar results [65]. It is postulated that such a reaction results from the fact that in some species, the cortical cell division does not activate under the influence of auxin, and as a result, the cells begin to lose contact with each other [61,66,67]. These results also indicated that the tissues that are located outside the vascular cylinder of the carrot hypocotyl are not involved in SE, and, therefore, may be an example of cells that are not competent for SE.

Similar to the results that were obtained in present studies, an analysis of the carrot hypocotyl showed that only the cells that originate from the divisions of the stele cells could generate cell lines and somatic embryos [65]. These insights confirm the results obtained in this study, which indicates that cells that are undergoing reprogramming come from the cells of the vascular cylinder, primarily the pericycle cells. Literature data have shown that the pericycle cells that are located on the xylem pole differ in their structure and ultrastructure from those that are located on the phloem pole [68,69]. Differences in the shape of the pericycle cells were also observed in this study, similar to those in the carrot root [70] and in contrast to what was observed in *Arabidopsis thaliana*, where the cells on the xylem side were shorter on the cross-section than those on the phloem side [68]. Such varied results may be due to species specificity.

The obtained results showed that the greatest changes in cells phenotype during the induction phase of SE concern the pericycle. According to numerous studies, the pericycle is a pluripotent tissue that is involved in processes such as initiating lateral root formation and secondary meristems [71,72]. The pericycle cells that are located on the xylem pole have also been shown to be involved in the SE in *Brassica oleracea* (L.) [73]. The results presented here show that under the influence of 2,4-D, both types of pericycle cells divide. Similar results have been described for in vitro cultures of *Daucus carota* [74], *Coffea canephora* [75], and *Acca sellowiana* [76]. The changes in the cytology of the cells in the explant, especially pericycle subjected to SE induction that was observed in the presented studies, state that these cells represent the reprogramming fate. Namely, cytological features such as dense cytoplasm, a high nucleus/cytoplasm ratio, an isodiametric cell shape, a spherically shaped nucleus with one or more nucleoli, small vacuoles, and thin cell walls are characteristics of pluripotent cells ([56] and literature therein). Such features have been observed in cells of different origins during the acquisition of embryogenic potential ([9,54,55,56,77] and literature therein). In the studies presented here, the changes in the cell wall composition were related to the observed cytological features for the cells that were undergoing reprogramming.

### 3.3. Spatio-Temporal Changes in Pectins as Markers of the State of Cell Differentiation

Pectins are an important component of plant cell walls that are associated with their mechanical properties [20,31]. They play a role in maintaining cell wall plasticity and cell adhesion, and they are also involved in cell growth [78] and differentiation [25]. Modifications of HG and a high degree of variability in the composition of the rhamnogalacturonan I (RG-I) side chains are often specific to individual cells or to a developmental program [79].

The presented results revealed that at the beginning of the culture, the galactan epitope (LM5) was present in the walls of all of the explant cells except for some of the anticlinal walls of the pericycle cells that were located on the xylem side. This may indicate that galactan-rich pectins are a negative marker of the cell position during normal hypocotyl pericycle development similar to AGPs and extensins for the pericycle cells of carrot and other species [45]. In the following days of culture, this epitope disappeared in the intensively dividing explant cells, this indicates that the walls of cells that were reprogrammed to a meristematic state are devoid of this epitope and that this pectic epitope is their negative marker. The question arises if observed changes can be interpreted as reprogramming pericycle cells from somatic to pluripotent state? Many studies have indicated that the pericycle is a pluripotent tissue [45]. Therefore, is it possible to consider a change in the direction of pericycle cell differentiation in the context of changing from a somatic to a pluripotent state? While this is rather not the case, the changes that were observed may indicate that it is an example of “meristem transdifferentiation” [72]. The most recent studies on Arabidopsis came to the conclusion that the pericycle cells are more pluripotent than was previously thought, at least for the pericycle cells that are located on the xylem pole [72]. The results obtained in the present study may be called “meristem transdifferentiation” [72], thus indicating that galactan-rich pectins are a negative marker of this process and also of the formative divisions that occurred during the induction of SE. However, it is undoubtedly a negative marker of the meristematic state of explant cells. The galactan side chains of RG-I are involved in maintaining cell stiffness [80], which may indicate that in cells that are reprogramming to a meristematic state, a decrease in this epitope is essential for the outgrowth of the clumps of proliferating cells.

It is worth paying attention to the detected presence of the LM5 epitope in separated cells. These cells were not involved in SE but nevertheless, this epitope can be used as a positive marker of detached cells that may have the potential to redifferentiate.

The obtained results showed that the distribution of the arabinan-rich pectins (LM6) was associated with the cytoplasmic compartments and with some walls of the cells with a meristematic character. This epitope can be assumed as a positive marker of cells that are reprogramming to a meristematic/pluripotent state. Literature data suggest that arabinans play an important role in cell adhesion, which has long been considered to be significant for the process of differentiation from meristematic cells [81]. Some studies have shown that the cells in the embryogenic clumps contain a low amount of LM6-reactive pectins [27,30,31,80,82,83]. It has been postulated that arabinans play the role of pectic plasticizers and that they are involved in maintaining cell wall flexibility [84]. The increased presence of this epitope in the cytoplasmic compartments and cell walls of the cells that were reprogramming to a meristematic state indicate the importance of wall flexibility in the proliferation process.

The degree of the methyl esterification of HG is recognized as a key determinant of plant development and organ formation, including processes such as cell division, growth, and adhesion [23,27]. The LM20 antibody detects an HG epitope with a high degree of methyl esterification [85]. In turn, the LM19 antibody detects an epitope of low methyl-esterified HG, whose level of esterification is less than 50% [85]. The epitope that is recognized by the LM20 antibody was present in all of the explant cell walls prior to their transfer to the induction medium as well as during the culture. No changes were observed in the occurrence of this epitope between the different explant cell types or culture time points, thus indicating that highly esterified pectins are an essential component of the cell walls of an explant, which is consistent with the literature data ([55] and literature therein). It also indicates that this epitope is not a marker of any changes in the cell reprogramming for the carrot hypocotyl explant during the induction phase of SE.

In contrast, there were significant differences in the distribution pattern of the epitope that is recognized by the LM19 antibody. At the beginning of the culture, there was a strong fluorescence signal in all of the explant cell walls, but with increasing culture time, it gradually disappeared, first in the periclinal walls of the dividing cells and next in all of the dividing cells. In the SE of *Q. suber* [86] and Cichorium [26], a small amount of de-esterified pectins was found in the pro-embryogenic masses. It is known that de-methylated HG residues can easily bind to calcium ions and, as a result, form a gel, which then leads to strong adhesion between the cells [23,25,87]. In the detached cells and those that were separating, this epitope was almost undetectable, which confirms its involvement in cell adhesion. To summarize, the presented results indicate that the de-esterification of pectins may accompany cells that are reprogramming to a meristematic/pluripotent state.

The obtained results demonstrated that the xylogalacturonan with a high degree of xylose (XGA) (recognized by the LM8 antibody) was present in the inner periclinal and fragments of the anticlinal walls of the cells that would detach. The presence of this epitope has been proven to be characteristic of cells that had already detached as well as in cells that were to be detached [88]. This phenomenon was also observed when testing the seed testa of legumes such as *Pisum sativum*, *Lupinus angustifolius,* and *Vigna radiata*. The XGA epitope was detected on the surface of the separating cells of the inner parenchyma [88]. In addition, the occurrence of the analyzed epitope was observed in the walls of the loosely adherent cells of the surface layers of the callus of two wheat varieties [89]. The involvement of the epitope that is recognized by the LM8 antibody in the process of cell separation has also been confirmed in developmental studies of the lateral and adventitious roots of *Solanum lycopersicum*, in which its presence was observed in the exfoliating cap cells [32]. The obtained results confirm the role of XGA in cell separation by “marking” the cells that will separate. Willats and colleagues [88] also observed the presence of this epitope in the cell walls that were loosely adhering to the cell aggregates that had formed in a *Daucus carota* suspension culture. The results presented here also indicate that this pectic epitope may be an early marker of cells that are predestined to detach.

### 3.4. Spatio-Temporal Changes of the AGPs as Markers of the State of Cell Differentiation

AGPs are involved in many developmental processes, not only in SE [54] but also in organ development [32], cell signaling ([90] and literature herein), cell wall expansion [34,91], and in many others ([92] and literature herein). Moreover, AGPs have a possibility to bind calcium ions, which means that it can participate in signal transduction [91]. Therefore, the spatio-temporal analysis of the AGPs presence/distribution in cell walls during the SE induction process is a good supplement to the existing knowledge.

The JIM4 antibody detects a common epitope for a set of cell membrane glycoproteins of carrot cells that are cultured in suspension culture as well as in the arabinogalactan proteins that are secreted by them [93]. The presence of this epitope in the carrot explant before the transfer to the induction medium was limited to the pericycle cells that were located on the xylem pole. A similar distribution pattern was observed during the root development of *Daucus carota* [45,93]. As was suggested by the authors, the presence of this epitope is associated with the position of the cells in the root, and in the present work, a comparable result was obtained. Casero and colleagues [45] showed that in the *Raphanus sativus* root, this epitope was located in pericycle on both the xylem and phloem sides, while in *Allium cepa* and *Pisum sativum*, the epitope was not found in the pericycle. By analyzing the above data and the results that are presented in this paper, it can be assumed that although the JIM4 antibody recognizes a specific cell type, its presence is species-specific.

Supposing that this epitope is associated with the cell position, can it then be assumed to be part of the cell specification markers? If so, the lack of this epitope in the explant cells during the induction phase of SE may indicate the loss of cell specificity. The lack of this epitope during SE induction was described (among others) for Agave [94], Brassica [95] or *T. nigrescens* [31], and Brachypodium embryogenic callus [30], which may confirm the above interpretation.

Before induction, the epitope that is recognized by the JIM8 antibody was detected to a small extent in the explant while in the pericycle cells on the xylem pole, it was absent, which indicates that this epitope is not constitutive for these cells. Toonen et al. [51] showed that the presence of the JIM8 epitope does not coincide with the ability of individual suspension cells to form embryos. The studies of McCabe et al. [52] showed that only cells without this epitope developed into somatic embryos. Thus, it appears that this epitope can be used as a negative marker of cells reprogramming to meristematic/pluripotent state. It is worth mentioning that the JIM8 epitope decorated the detached cells, thus it can be assumed to be a positive marker of separation.

The JIM13 antibody detects the chain in the carbohydrate part of the arabinogalactan proteins [96]. In the carrot hypocotyl, before the transfer to the induction medium, the epitope was present in all of the cells, but during the culture, it disappeared in the dividing cells. Similar results were obtained for Arabidopsis [54] and *T. nigrescens* [31]. The presented results indicate that this epitope can be used as a negative marker of cell reprogramming to meristematic/pluripotent state and a positive marker of cells that do not change their direction of differentiation. Moreover, this AGP epitope is a perfect marker of the cell separation process as well as a detached cell.

To date, the epitope structure in the arabinogalactan proteins, which is recognized by the JIM16 antibody [96], has not been specified. In the presented studies, the punctate signals generated by this antibody was almost absent. However, with the increasing duration of the culture, the appearance of the signal increased in the cells with dense cytoplasm that were dividing. This epitope was primarily located in the cytoplasmic compartment, similar as it has been observed during *A. thaliana* SE [54], *Mussa* spp. [38], or wheat [41]. The presence of this epitope has also been observed in the dividing cells at an early stage of adventitious root formation in *Solanum lycopersicum* [32] and in the Brachypodium embryogenic callus [30]. The results obtained in the present work and those that were presented by Sala and colleagues [32] suggest the participation of this epitope during intensive cell division and characterize the cells that gain pluripotency. This indicates that the JIM16 “decoration” marks cells that are reprogramming their fate.

During the culture on the induction medium, there was a clear fluorescence signal of the LM2 antibody in the cell walls and cytoplasmic compartments of the dividing cells with dense cytoplasm. A similar pattern of the distribution of this epitope has been observed in the actively dividing cells during *Triticum aestivum* organogenesis [41]. In the embryogenic callus of Brachypodium, this epitope was detected in the embryogenic cells [30] and during the SE of *T. nigresens* [31], however, in the Arabidopsis explant [54] and in *Actinidia arguta* [55], it was not detected in the cells that were changing their fate. The results indicate that during the induction phase in a carrot explant, this epitope can be considered to be a marker of the cells that are reprogramming their fate.

### 3.5. Spatio-Temporal Changes of the Extensins as Markers of the State of Cell Differentiation

Proteins that are rich in hydroxyproline (HRGP), to which extensins are included, are important in many aspects of plant development and growth [97,98]. However, as was emphasized by Xu and colleagues [48], little is known about the location and function of extensins during the somatic embryogenesis of higher plants or during the cell reprogramming. The JIM11 and JIM20 antibodies recognize the glycoprotein oligosaccharide epitope in the side chain, and the JIM20 antibody is thought to recognize this chain in extensins, while JIM11 does this in the extensins and lectins [47]. The epitope that is recognized by the JIM12 antibody contains a protein component in the glycoprotein region [47]. In the present work, only the epitope that is recognized by JIM11 can be postulated as being a marker of the detached cells. In the Brachypodium callus [30] and in the *Actinidia arguta* callus [55], this epitope was detected in the intercellular spaces. This result may indicate the involvement of extensins in increasing the strength of the walls, which was also found, among others, by Knox [19].

## 4. Materials and Methods

### 4.1. Plant material and Culture Condition

The carrot seeds (*Daucus carota* L. ‘Flakkese 2 Trophy Zif’) were surface sterilized in a 75% aqueous ethanol solution for 15 min, and then in an undiluted commercially available bleaching agent containing sodium hypochlorite that had been supplemented with Triton X-100 (100 mL/10 mL) for 15 min. The seeds were washed 3 times for 2 min in sterile distilled water, then dried on sterile paper and transferred to a solid B5 medium [99]. The culture was carried out at 24 ± 1 °C in the dark for 10 days.

### 4.2. In Vitro Culture

The hypocotyls from etiolated carrot seedlings were used as the explant. The hypocotyls were cut into segments of about 1 cm and then transferred onto a solid E5 induction medium containing 2 μM 2,4-dichlorophenoxyacetic acid (2,4-D; [99]) for 18 days (temperature 24 ± 1 °C; photoperiod 16/8 h, light intensity 20 μM m^−2^ s^−1^).

Histological and immunohistological analyses were performed on at least 3 hypocotyls at each time point (see below). This experiment was repeated twice. Day 0 was a hypocotyl that had been cut from a seedling, which was transferred to an induction medium and was essentially a control. The analyses were compared to the characteristic features of hypocotyls at the beginning of the experiment (day 0).

### 4.3. Histochemistry and Immunohistochemistry

Fragments of the etiolated hypocotyls from seedlings after 10 days on the B5 medium (time point 0) and at subsequent time points on the induction medium (after 1, 2, 3, 4, 5, 10, and 18 days) were fixed according to the procedures that were described earlier [32,100]. Briefly, for the immunohistochemical analyses, the samples were fixed in a solution of 4% paraformaldehyde (PFA) and 1% glutaraldehyde in phosphate-buffered saline (PBS) at pH 7.0, washed 3 times in PBS, dehydrated in a graded ethanol series, and embedded in LR White resin. The samples were cut into 1.5 µm thick cross-sections using a Leica EM UC6 ultramicrotome. Sections were collected on microscopic slides covered with poly-l-lysine (Menzel Gläser, Braunscheig, Germany).

For the immunolabelling procedure, the sections were processed, as was described earlier [32,100]. The used primary rat monoclonal antibodies (Plant Probes, Leeds, UK) are listed in Table 2. As a secondary antibody, the AlexaFluor 488 goat anti-rat was used (Cat. No. 112-545-003) (Jackson ImmunoResearch Laboratories, West Grove, PA, USA). The negative controls were prepared by omitting the primary antibody. The sections were counterstained with 0.01% (*w*/*v*) Calcofluor White (Fluorescent Brightener 28; Cat. No. F3543, Sigma-Aldrich, St. Louis, MO, USA) in PBS for 10 min for visualization of the cell walls in the sections.

For the histological analysis, the samples were fixed in 5% (*w*/*v*) GA in 0.1 M PBS (pH 7.2) and the sections were stained with a 0.05% water solution of toluidine blue O (TBO) for 5 min [54]. The sections were also stained with DAPI (4′,6-diamidino-2-phenylindole; Invitrogen Inc.; 5 min at RT—room temperature; 2 µg/mL in PBS) and mounted in Vectashield (Vector Laboratories, Peterborough, United Kingdom).

The observations and photo-documentation were performed using a Nikon ECLIPSE Ni-U fluorescence microscope equipped with a Nikon Digital DS-Fi1-U3 digital camera and an Olympus BX60 bright field equipped with a CCD matrix digital camera. To observe the results of immunostaining, the filter sets for Alexa Fluor 488 were used (excitation filter 450–490, barrier filter BA520) and for DAPI and calcofluor (excitation filter 330–380, barrier filter BA420). Photo editing: Photos were edited in PhotoScape v3.7 and Corel PaintShop Pro X6, scale was used, and plates were created in CorelDRAW X7 v.17 (all of the programs are legally available; brightness and contrast were adjusted).

## 5. Conclusions

Based on the obtained results, the most important conclusions that can be drawn are: (1) The pectin rich in arabinan side-chains epitopes (LM6) and arabinogalactan protein with carbohydrate epitope containing β-linked GlcA (LM2) are positive markers of cells reprogramming to the meristematic/pluripotent state; (2) the pectin rich in galactan side-chains epitopes (LM5) and unmethyl-esterified as well as arabinogalactan proteins containing the LM19 (pectic), JIM8 and JIM13 epitopes (AGPs) are negative markers of cells reprogramming to the meristematic/pluripotent state; (3) XGAwith high degree of xylose (LM8), AGPs with JIM8, JIM13, LM2 (AGPs) epitopes and JIM11 (extensin) epitopes are positive markers, but LM6 (pectic) is the negative marker of cells undergoing detachment, which are not involved in the induction of somatic embryogenesis; (4) AGPs with JIM4 epitope is a positive marker, but LM5 (pectic), AGPs with JIM8; JIM13 and LM2 epitopes are negative markers for pericycle cells on the xylem pole; (5) methyl- and un-methyl esterified pectins (LM19, LM20), AGPs with JIM13 and LM2 epitopes are positive constitutive wall components, but LM6, LM8 (pectic), AGPs with JIM4, JIM8, JIM16, extensins (JIM11, JIM12, JIM20) are not a constitutive wall components, and (6) the extensins do not contribute much to the induction of somatic embryogenesis in carrot in which the cell fate is changed.

## Figures and Tables

**Table 1:** The determined positive and negative markers of the cellular events that occurred during the changes of the direction of cell differentiation in a *Daucus carota* (L.) hypocotyl being cultured on the induction medium.

	Cells Reprogramming to the Meristematic/Pluripotent State	Cells Undergoing Separation	Separated Cells	Formative Divisions	Constitutive Wall Component	Cell Position Marker
**Positive Marker**	LM6, LM2	LM8, JIM8, JIM13, LM2, JIM11	LM5, JIM8, JIM13, LM2	LM6, JIM16, LM2, JIM13	LM19, LM5, LM20, JIM13, LM2	JIM4, for pericycle cells on xylem pole
**Negative Marker**	LM5, JIM8, JIM13	LM6	LM6	LM5	LM6, JIM4, JIM8, JIM16, JIM12, JIM20	LM5, JIM8; JIM13, LM2 for pericycle cells on xylem pole

**Table 2:** List of the primary rat monoclonal antibodies that were used in the current study.

Antibody	Epitope	References
Pectins		
LM5	linear tetrasaccharide in (1–4)-β-D-galactans (RG I side chain)	[101]
LM6	linear pentasaccharide in (1–5)-α-l-arabinans (RG I side chain)	[102]
LM8	xylogalacturonan domain in HG	[88]
LM19	non-methyl-esterified, partially methyl-esterified HG	[85]
LM20	methyl-esterified HG	[85]
Extensins		
JIM11	Extensin/ HRGP glycoprotein	[47]
JIM12	Extensin/ HRGP glycoprotein	[47]
JIM20	Extensin/ HRGP glycoprotein	[47]
AGPs		
LM2	Arabinogalactan protein, carbohydrate epitope containing β -linked GlcA	[103]
JIM4	Arabinogalactan glycoprotein, βGlcA-(1,3)- α GalA-(1,2)-Rha	[93,96]
JIM8	Arabinogalactan	[104]
JIM13	Arabinogalactan/Arabinogalactan protein, carbohydrate epitope (β)GlcA1->3(α)GalA1->2Rha	[105]
JIM16	Arabinogalactan/Arabinogalactan protein	[105]

## Supplementary Materials

The following are available online at https://www.mdpi.com/1422-0067/21/21/8126/s1.

## Abbreviations

AGP	Arabinogalactan protein
DAPI	4′,6-diamidino-2-phenylindole
HG	Homogalacturonan
RG-I	Ramnogalacturonan I
HRGP	Hydroxyproline-rich glycoproteins
SE	Somatic embryogenesis
XGA	Xylogalacturonan
