# Supplementary material for: Cell Wall Composition as a Marker of the Reprogramming of the Cell Fate on the Example of a Daucus carota (L.) Hypocotyl in Which Somatic Embryogenesis Was Induced
Source: Int J Mol Sci. 2020 Oct 30;21(21):8126. doi: 10.3390/ijms21218126 (PMC7662930; doi:10.3390/ijms21218126)
Supplement: Supplementary file 1 [file ijms-21-08126-s001.pdf]

Supplementary Materials

Cell Wall Composition as a Marker of the Reprogramming of the Cell Fate on the Example of a *Daucus carota* (L.) Hypocotyl in Which Somatic Embryogenesis Was Induced

Michał Kuczak [1] and Ewa Kurczyńska [2,*]

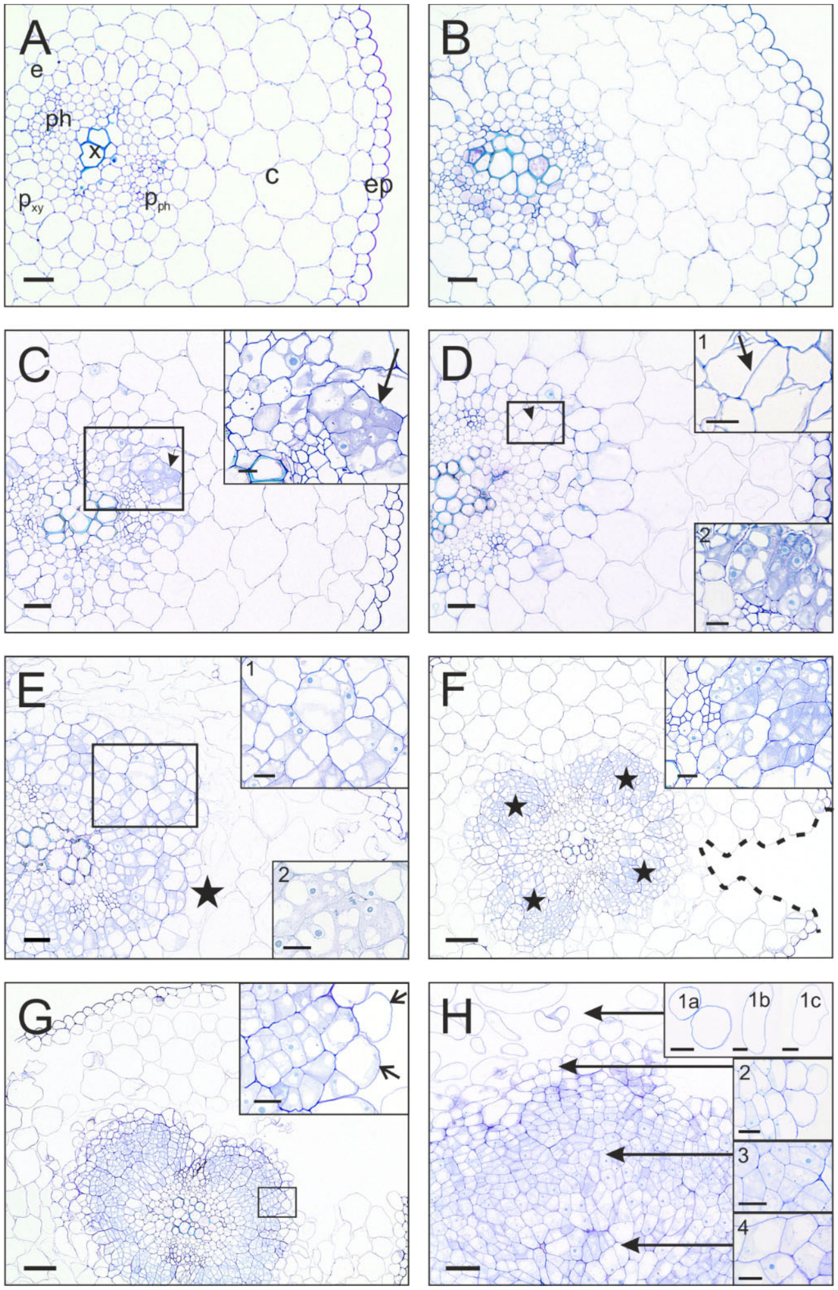

Figure S1A. Representative explant histology (cross sections) at different time points of the culture. (**A**) Histology of the explant at the beginning of culture and (**B**) after one day on induction medium. (**C**) After two days of the culture the pericycle cell divisions that led to the formation of cells with a meristematic character were observed (C and inset – arrow, D and inset 2). (**D**) Anticlinal divisions of the pericycle cells that were located on the xylem pole were detected after four days (inset 1 – arrow). (**E**) At the next day of the culture abundant divisions of the pericycle cells and the more advanced process of the production of meristematic cells (inset 1), mitoses (inset 2) and the simultaneous process of separating the cortex (area marked by a star) were observed. (**F**) Four clear poles with cells with a meristematic character (stars) and cell complexes (inset, notice the much darker blue and thicker walls at the borders of the cell complexes; separation of the cortex and epidermis marked with a dashed line) after five days on induction medium were well visible. (**G**) At the tenth day, highly vacuolated cells appeared at the explant surface (inset – arrows). (**H**) At the end of culture, the occurrence of: inset 1a – round-shaped cells; inset 1b – rod-shaped cells; inset 1c – variously shaped cells; inset 2 – cells on the stele border; inset 3 – intensively dividing cells with dense cytoplasm and several small vacuoles; inset 4 – intensively dividing cells with dense cytoplasm and one large vacuole (arrows point to areas showed on insets) were detected; (ep – epidermis, c – cortex; p_{xy} – pericycle on the xylem pole; p_{ph} – pericycle on the phloem pole; ph phloem, x – xylem; e – endodermis). All of the sections were stained with TBO. Scale bars: A-E -20 μm, insets – 10 μm; F-H – 50 μm, insets – 10 μm.

Table S1. Summarized results of immunolabeling during the culture period and in different tissue/cells

Culture duration (d)	0			2-3		4-5		10			18		
	**Whole explant**	**Pericycle**	**Cortex**	**Whole explant**	**Meristematic cells**	**Whole explant**	**Meristematic cells**	**Whole explant**	**Meristematic cells**	**Detached cells**	**Whole explant**	**Meristematic cells**	**Detached cells**
**Pectins**													
**LM5**	++	±, on xylem pole	++	++	--	++	--	++	-	++	--	--	++
**LM6**	--	--	--	+	+, also in c	+	++, also in c	+	±	±	±	±	--
**LM8**	--	--	--	--	--	--	--	--	-	++, surface cells	--	--	++, surface cells
**LM19**	+	+	+	++	++	++, and in intercellular spaces	--	+	++	±	+	--	±
**LM20**	++	++	++	++	++	++	++	++	++	++	++	++	++
**AGP**													
**LM2**	+	-- only on xylem pole	+	+	+	--, apart from surface cells	+, c	++	++	±	--	--	± and surface cells
**JIM4**	--	+ on xylem pole	--	--	--	--	--	--	--	--	--	--	--
**JIM8**	++	-- if present only on xylem pole	+	±	±	±	--	+	--	+	--	--	++
**JIM13**	++	++, but – on the xylem pole	++	+	+, but lack on the xylem pole	+	--	+	--	++	--	--	++
**JIM16**	--	--	--	--	--	+	+	--	--	--	--	--	--
**Extensins**													
**JIM11**	--	--	--	--	--	--	-	--	--	±	--	--	±
**JIM12**	-- (± - xylem elements)	--	--	--	--	--	--	--	--	--	--	--	--
**JIM20**	-- (± - xylem elements; intercellular spaces)	--	--	--	--	--	--	--	--	--	--	--	--

The intensity of labelling was evaluated (based on visual estimation) as: -- no labelling, ± weak labelling, + moderate labelling, and ++ strong labelling (according to Bárány et al., 2010 and Potocka et al., 2018; c=means that also in cytoplasmic compartments epitope was detected; w=wall; d=days; from the 2nd day of the culture the pericycle is no longer mentioned as meristematic cells appeared; detached cells = cells separated from the explant).

Table S2. Quantification of the identified epitopes of pectins, AGPs and extensins in individual cells/tissues during the culture.

**Culture duration (d)**	**0**			**2-3**		**4-5**		**10**			**18**		
	**Most of the explant cells**	**Pericycle**	**Cortex**	**Most of the explant cells**	**Meristematic cells**	**Most of the explant cells**	**Meristematic cells**	**Most of the explant cells**	**Meristematic cells**	**Detached cells**	**Most of the explant cells**	**Meristematic cells**	**Detached cells**
**Pectins**													
**LM5**	28*/30**<br>84%^	29*/30**<br>96.6%	30*/30**<br>100%	27*/30**<br>90%	0*/30**<br>0%	30*/30**<br>100%	2*/30**<br>6.6%	19*/30**<br>63.3%	0*/30**<br>0%	30*/30**<br>100%	0*/30**<br>0%	0*/30**<br>0%	30*/30**<br>100%
**LM6**	0*/30**<br>0%	0*/30**<br>0%	0*/30**<br>0%	29*/30**<br>96.6%	29*/30**<br>96.6%	29*/30**<br>96.6%	29*/30**<br>96.6%	0*/30**<br>0%	28*/30**<br>93.3%	0*/30**<br>0%	11*/30**<br>36.6%	29*/30**<br>96.6%	0*/30**<br>0%
**LM8**	0*/30**<br>0%	0*/30**<br>0%	0*/30**<br>0%	0*/30**<br>0%	0*/30**<br>0%	0*/30**<br>0%	0*/30**<br>0%	2*/30**<br>6.6%	0*/30**<br>0%	30*/30**<br>100%	0*/30**<br>0%	0*/30**<br>0%	30*/30**<br>100%
**LM19**	29*/30**<br>96.6%^	30*/30**<br>100%	30*/30**<br>100%	29*/30**<br>96.6%	29*/30**<br>96.6%	26*/30**<br>86.6%	7*/30**<br>23.3%	27*/30**<br>90%	5*/30**<br>16.6%	5*/30**<br>16.6%	25*/30**<br>83.3%	4*/30**<br>13.3%	5*/30**<br>16.6%
**LM20**	30*/30**<br>100%^	30*/30**<br>100%^	30*/30**<br>100%^	30*/30**<br>100%^	30*/30**<br>100%^	30*/30**<br>100%^	30*/30**<br>100%^	30*/30**<br>100%^	30*/30**<br>100%^	30*/30**<br>100%^	30*/30**<br>100%^	30*/30**<br>100%^	30*/30**<br>100%^
**AGP**													
**LM2**	29*/30**<br>96.6%	0*/30**<br>0%	30*/30**<br>100%	25*/30**<br>83.3%	27*/30**<br>90%	29*/30**<br>96.6%	27*/30**<br>90%	24*/30**<br>80%	30*/30**<br>100%	2*/30**<br>6.6%	24*/30**<br>80%	26*/30**<br>86.7%	18*/30**<br>63.3%
**JIM4**	0*/30**<br>0%	18*/30**<br>60%#	1*/30**<br>3.3%	0*/30**<br>0%	0*/30**<br>0%	0*/30**<br>0%	0*/30**<br>0%	0*/30**<br>0%	0*/30**<br>0%	0*/30**<br>0%	0*/30**<br>0%	0*/30**<br>0%	0*/30**<br>0%
**JIM8**	27*/30**<br>90%	0*/30**<br>0%#	28*/30**<br>93.3%	16*30**<br>53.3%	2*/30**<br>6.6%	12*/30**<br>40%	1*/30**<br>3.3%	14*/30<br>46.6%	0*/30**<br>0%	29*/30**<br>96.6%	0*/30**<br>0%	0*/30**<br>0%	30*/30**<br>100%
**JIM13**	30*/30**<br>100%^	30*/30**<br>100%	30*/30**<br>100%	30*/30**<br>100%	30*/30**<br>100%	24*/30**<br>80%	2*/30**<br>6.6%	22*/30**<br>73.3%	0*/30**<br>0%	28*/30**<br>93.3%	27*/30**<br>90%	0*/30**<br>0%	30*/30**<br>100%
**JIM16**	0*/30**<br>0%	0*/30**<br>0%	0*/30**<br>0%	0*/30**<br>0%	0*/30**<br>0%	11*/30**<br>36.6%	28*/30**<br>93.3%	9*/30**<br>30%	3*/30**<br>10%	8*/30**<br>26.6%	0*/30**<br>100%	0*/30**<br>0%	26*/30**<br>86.6% &
**Extensins**													
**JIM11**	0*/30**<br>0%	0*/30**<br>0%	0*/30**<br>0%	0*/30**<br>0%	0*/30**<br>0%	0*/30**<br>0%	0*/30**<br>0%	0*/30**<br>0%	0*/30**<br>0%	0*/30**<br>0%	0*/30**<br>0%	0*/30**<br>0%	30*/30**<br>100%
**JIM12**	0*/30**<br>0%	0*/30**<br>0%	0*/30**<br>0%	0*/30**<br>0%	0*/30**<br>0%	0*/30**<br>0%	0*/30**<br>0%	0*/30**<br>0%	0*/30**<br>0%	0*/30**<br>0%	0*/30**<br>0%	0*/30**<br>0%	0*/30**<br>0%
**JIM20**	0*/30**<br>0%	0*/30**<br>0%	0*/30**<br>0%	0*/30**<br>0%	0*/30**<br>0%	0*/30**<br>0%	0*/30**<br>0%	0*/30**<br>0%	0*/30**<br>0%	0*/30**<br>0%	0*/30**<br>0%	0*/30**<br>0%	0*/30**<br>0%

*Number of sections with detected epitope, **Number of tested sections. % - it is a percentage from ratio of number of sections exhibited epitope presence to the number of all analysed sections. The numbers quoted relate to all sections analyzed (all replicates; no divided for each replicate; data from 3 replicates in each biological repetition; 30 sections were analyzed at each time point and for each epitope/tissue/cell; # indicates the value for pericycle cells; & indicates that also in surface cells epitope was detected; ^ indicates the occurrence of the epitope in whole explant).

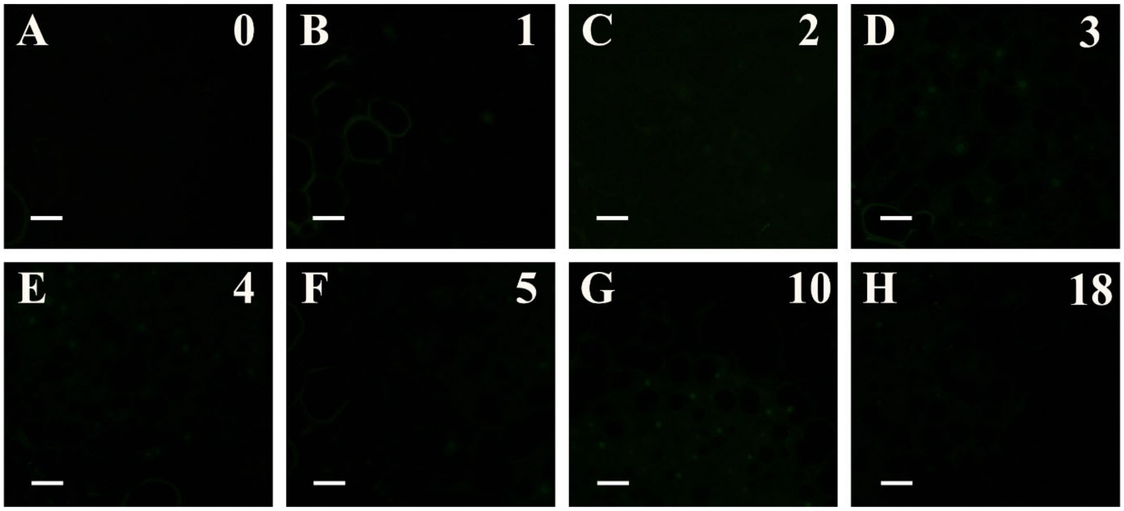

Figure S1B. Representative cross sections after control reactions curried out without the primary antibody (LM5) against pectic epitope (numbers indicate the days of culture; the same results were obtained for all other epitopes). Scale bars = 10 μm.

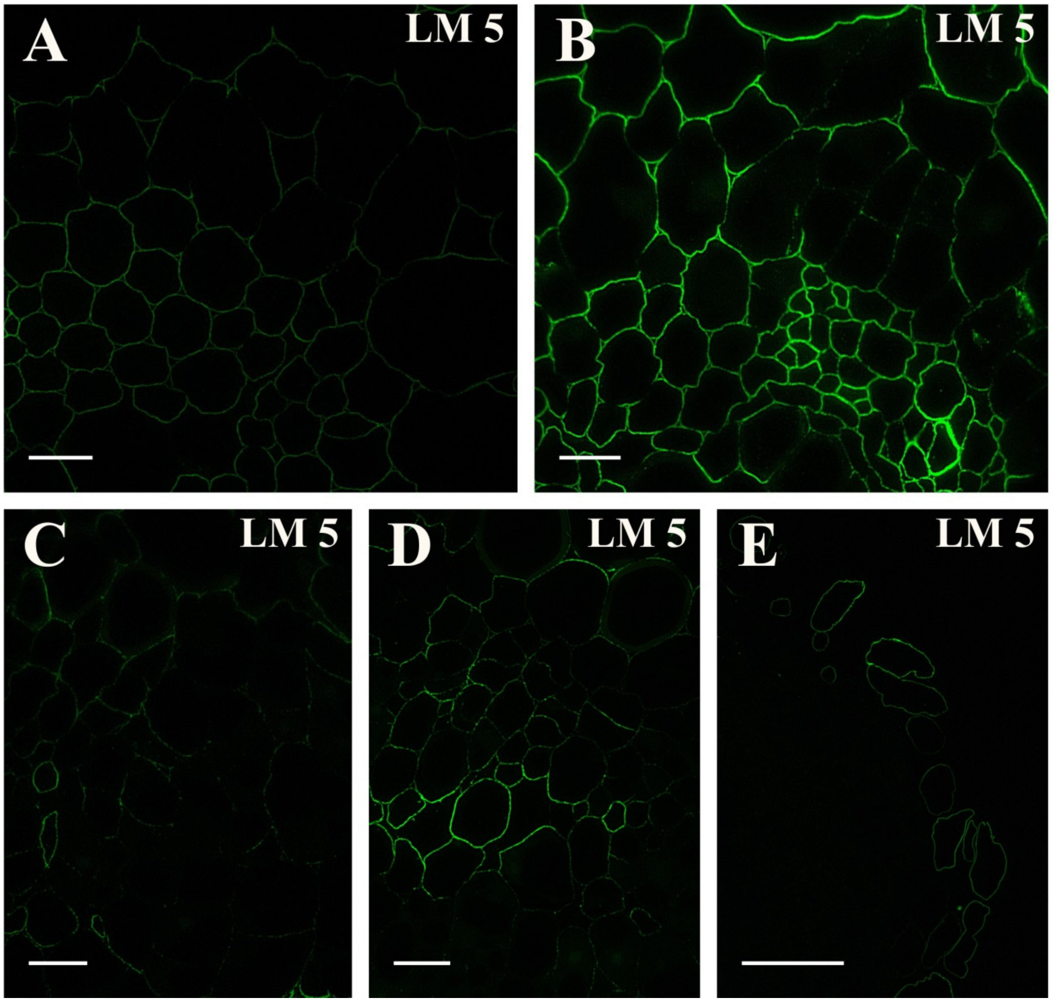

Figure S2A. Representative distribution of the pectic epitope that is recognised by the LM5 antibody on particular days of the culture obtained in the second replication. Presence of the signal at the beginning (**A**), after two days (**B**), four days (**C**), five days (**D**) and 18 days (**E**) of the culture is visible. Scale bars: A-D – 10 μM; E – 50 μm.

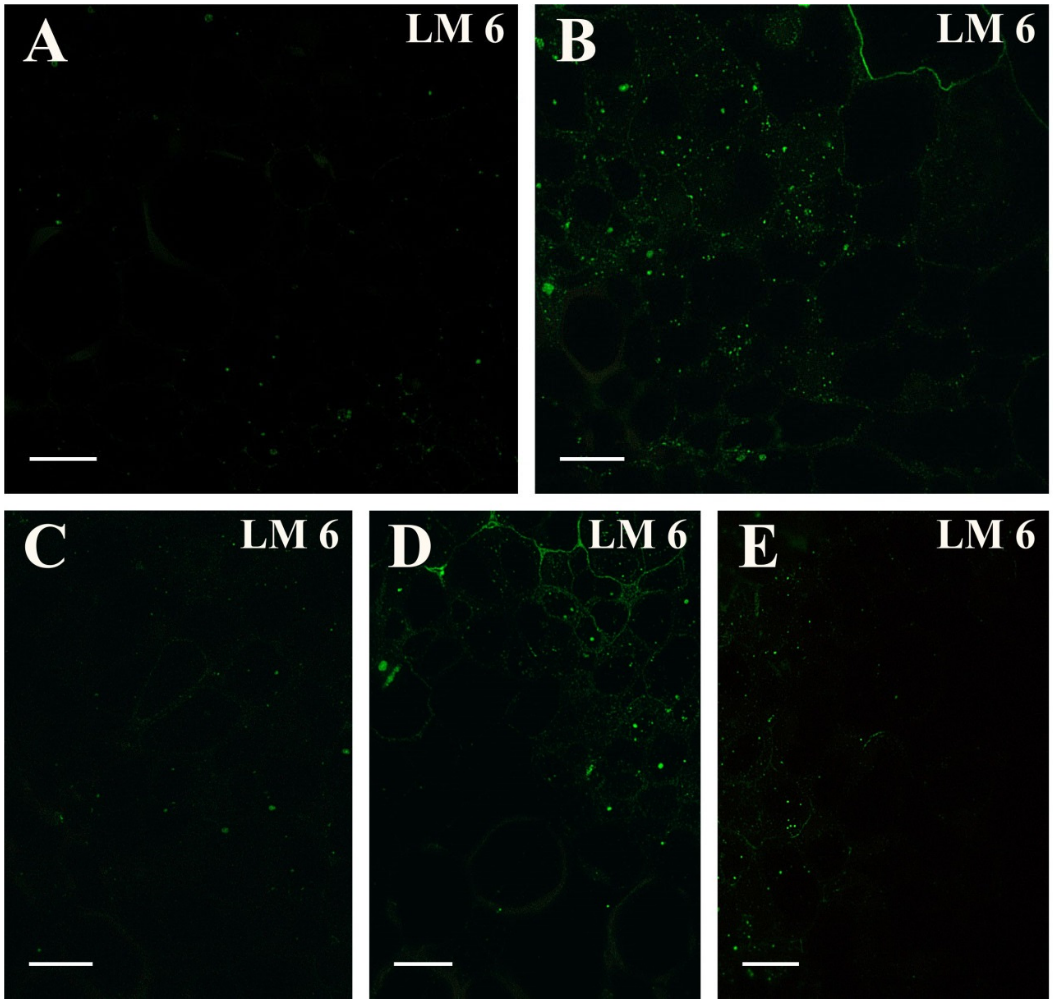

Figure S2B. Distribution of the pectic epitope that is recognised by the LM6 antibody detected in the second replication. At the beginning of the culture (**A**) signal was almost not present. In the following days of the culture the appearance of signal in dividing cells was observed [(**B**), four days (**C**), five days (**D**) and 18 days (**E**) of the culture]. Scale bars: 10 μm.

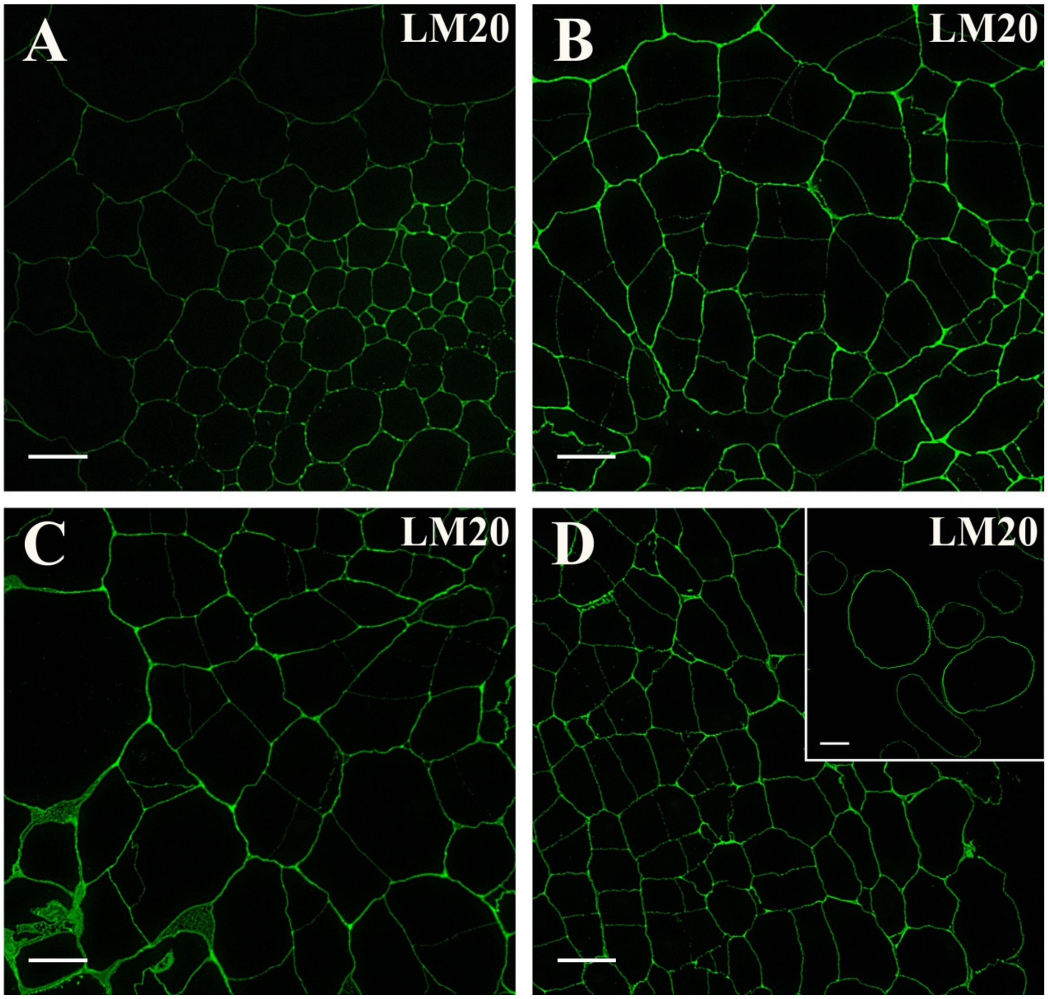

Figure S2C. Representative distribution of the pectic epitope that is recognised by the LM20 antibody observed in second replication. Regardless of the time of culture, a clear signal was present in the walls of all cells of the explant: from the beginning of the culture (**A**), after four days (**B**), five days (**C**) and 18 days (**D**) of the culture. Enlargement of the separated cells (D – inset). Scale bars: 10 µm.

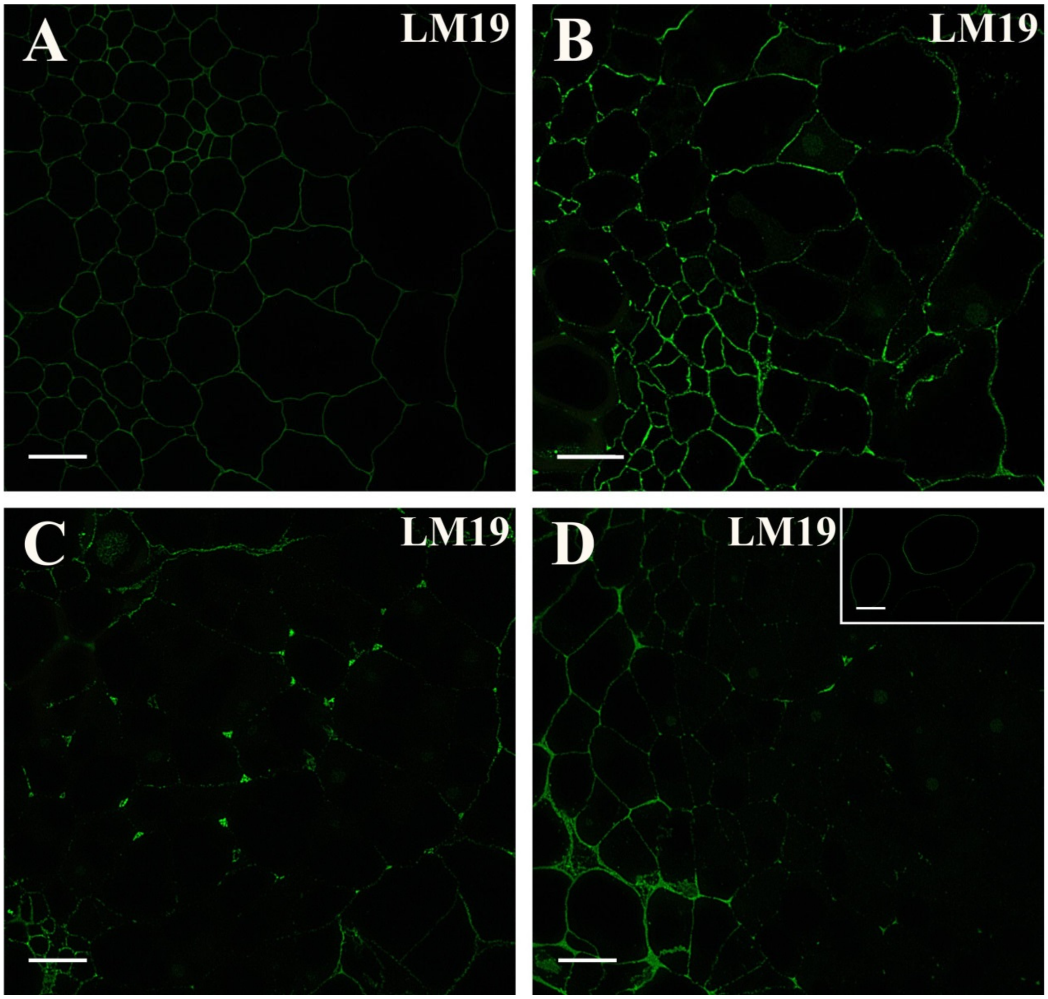

Figure S2D. Presence of the pectic epitope that is recognised by the LM19 antibody within the explant, obtained during the second repetition. Similar distribution of this epitope as in Fig. 4 was observed [(**A**) at the start of the culture, (**B**) after two days, four days (**C**) and 18 days (**D**) of the culture]. Enlargement of the separated cells (D – inset). Scale bars: 10 μm.

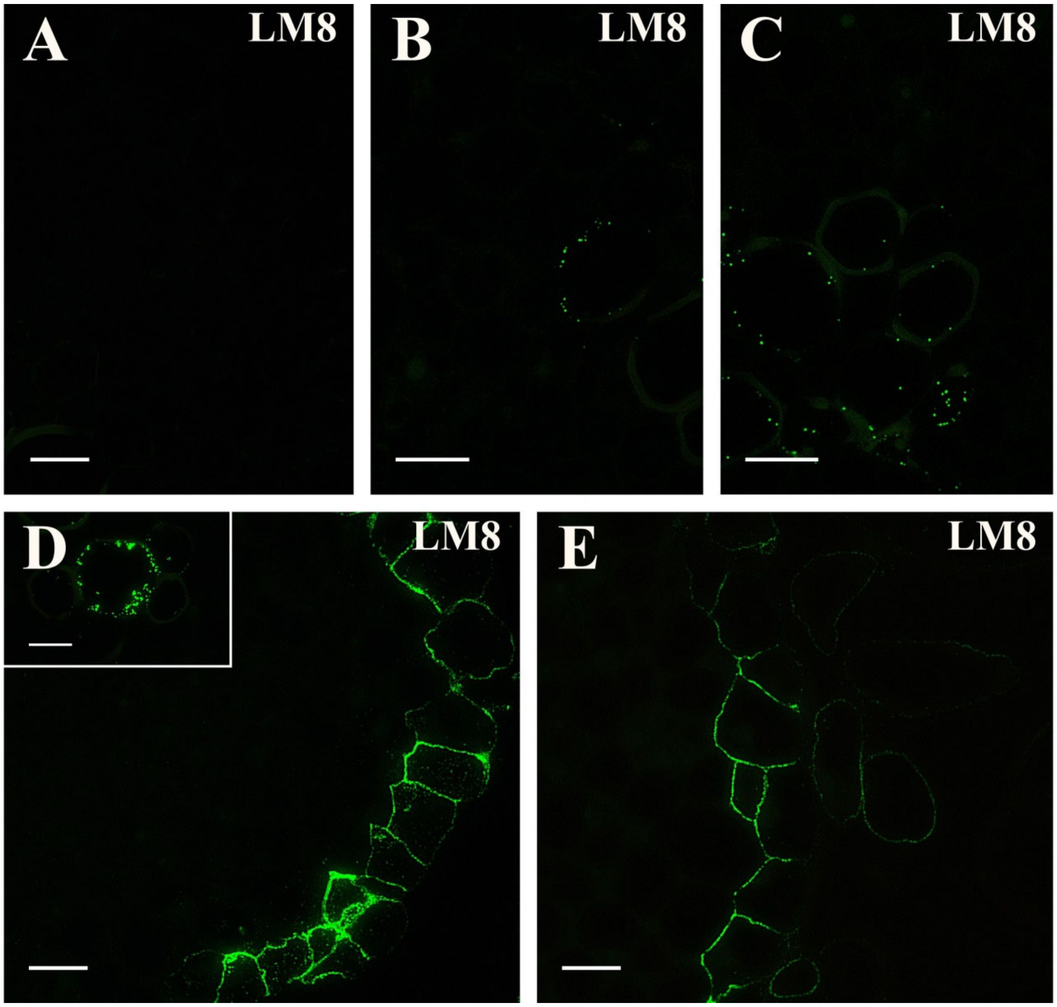

Figure S2E. Pectic epitope that is recognised by the LM8 antibody was not detected at the beginning of the culture (**A**). After following days (**B**, **C** after two and four days respectively) signal was detected only in vessel elements. (**D**). At the tenth day signal marks the cells predestined to separation. (**E**) At the end of the culture (18 day) signal was also present in detached cells. Enlargement of the differentiating vessels (D – inset). Presented results have been obtained during the second replication. Scale bars: 10 μm.

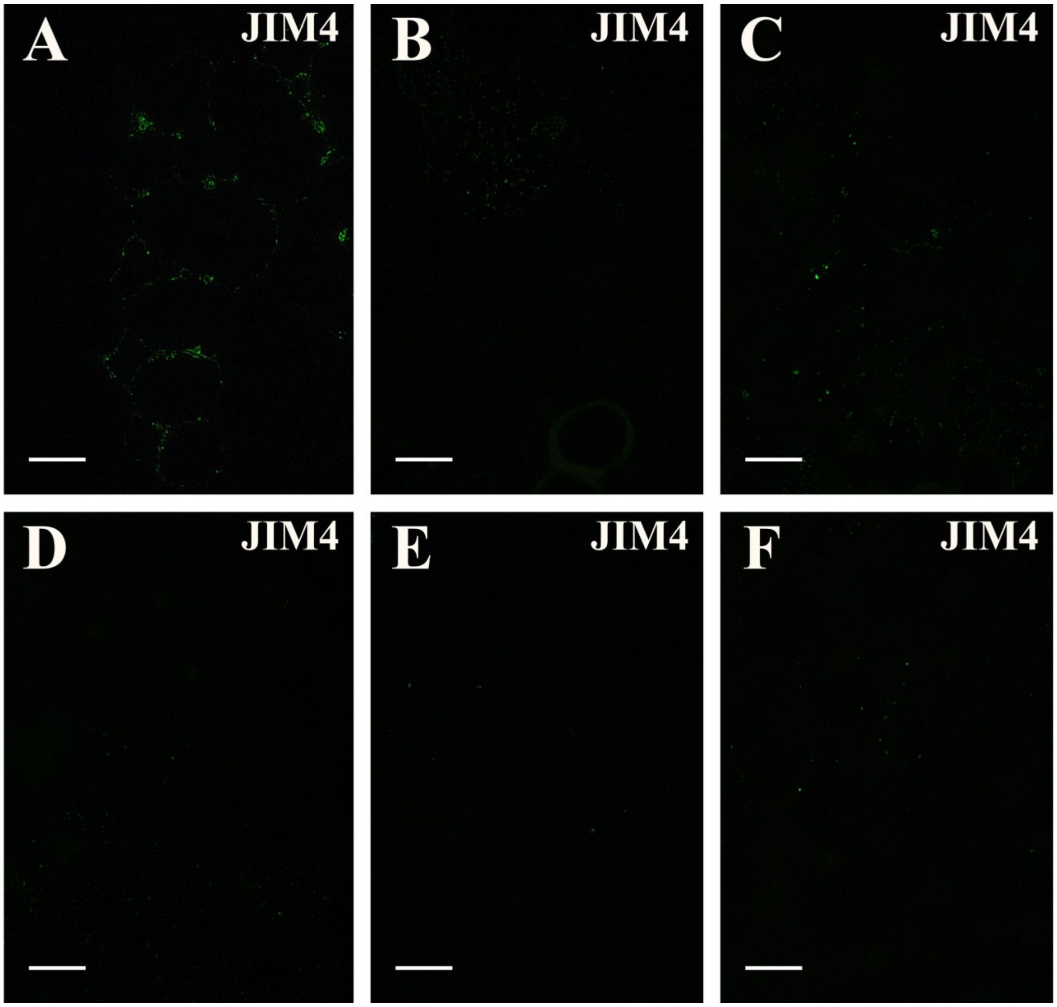

Figure S2F. Distribution of the AGP epitope that is recognised by the JIM4 antibody observed in explant tissues during the second repetition. (**A**) At the beginning of the culture signal was detected only in some pericycle cells. In the next days (**B** – two days; **C** - four days; **D** – five days; **E** – ten days and **F** - 18 days) signal disappeared. Scale bars: 10 μm.

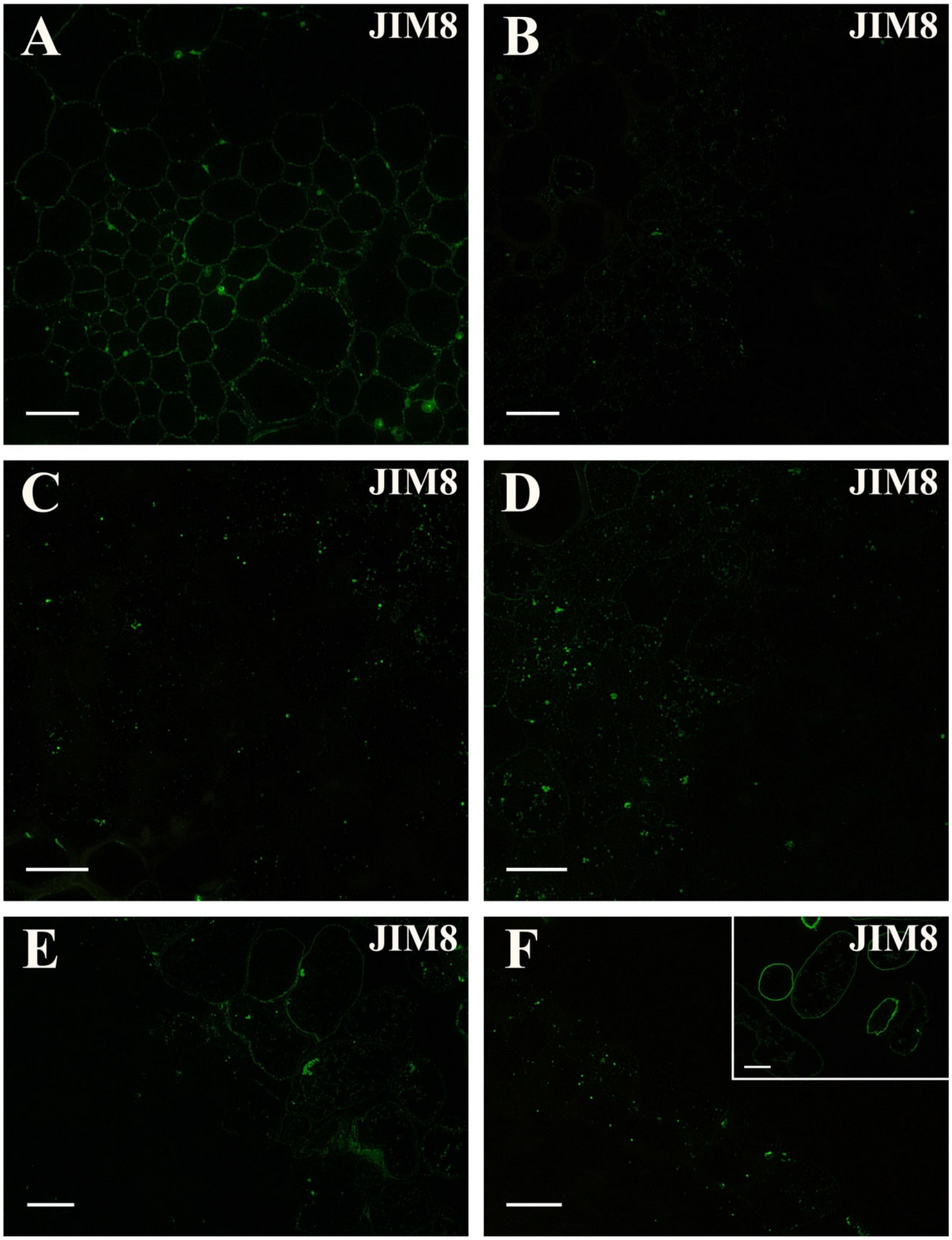

Figure S2G. Representative distribution of the AGP epitope that is recognised by the JIM8 antibody detected during the second repetition. Presence of the signal was similar to that shown on Fig. 7 [(**A**) – culture beginning; after three days (**B**), four days (**C**), five days (**D**), ten days (**E**) and 18 days (**F**) of the culture]. Enlargement of the separated cells (F – inset). Scale bars: 10 μm.

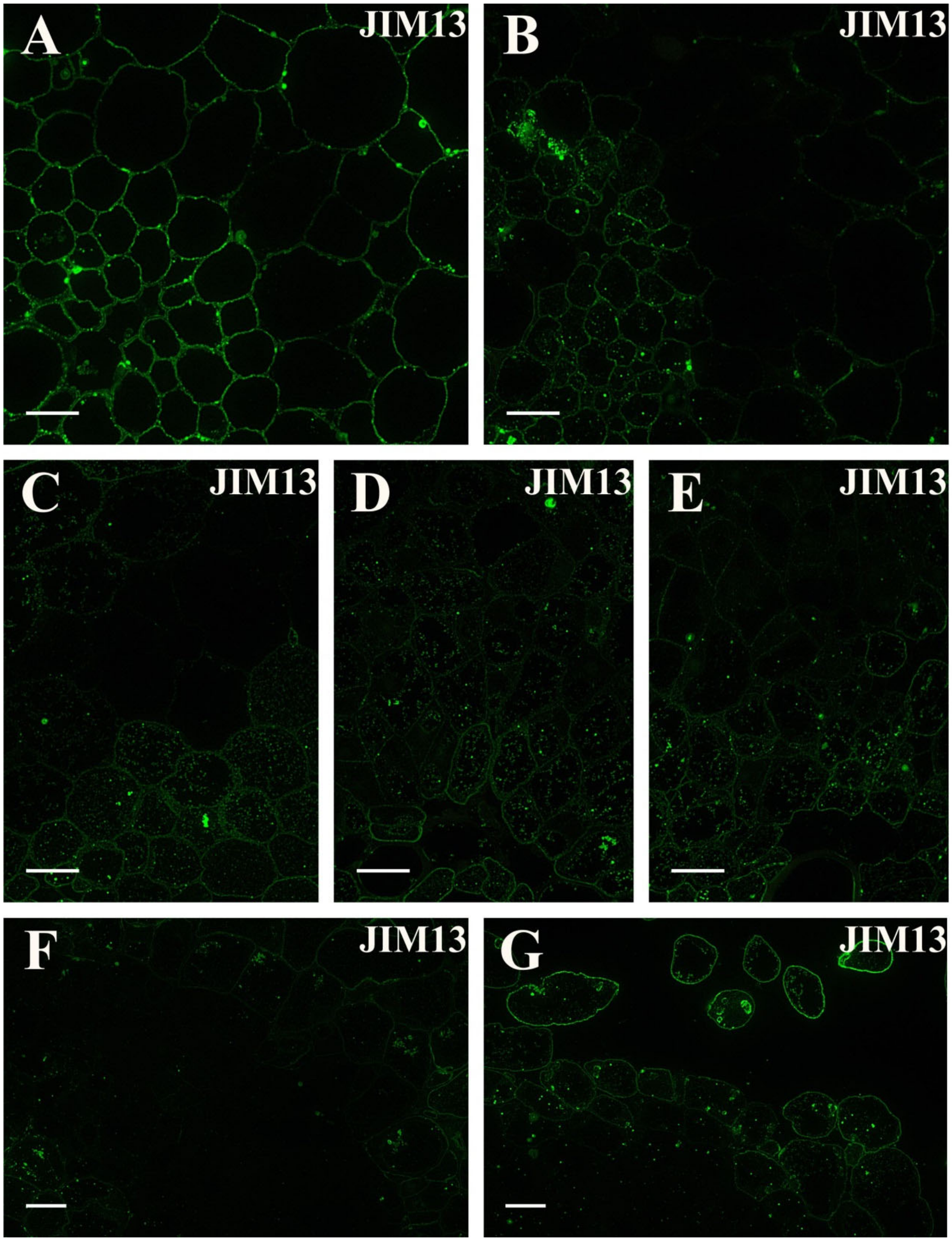

Figure S2H. The AGP epitope that is recognised by the JIM13 antibody was well visible at the beginning of the culture (**A**). During the first two days (**B** – one day; **C** - two days) lack of signal marks the pericycle cells from the xylem pole. During following days of the culture (**D** - four days; **E** - five days) signal was detected in dividing cells. From the tenth day (**F** – tenth day, **G** – 18th day) clear signal was present in the separated cells but progressive loss of signal in the dividing cells was observed. All photos were obtained during the second repetition. Scale bars: 10 μm.

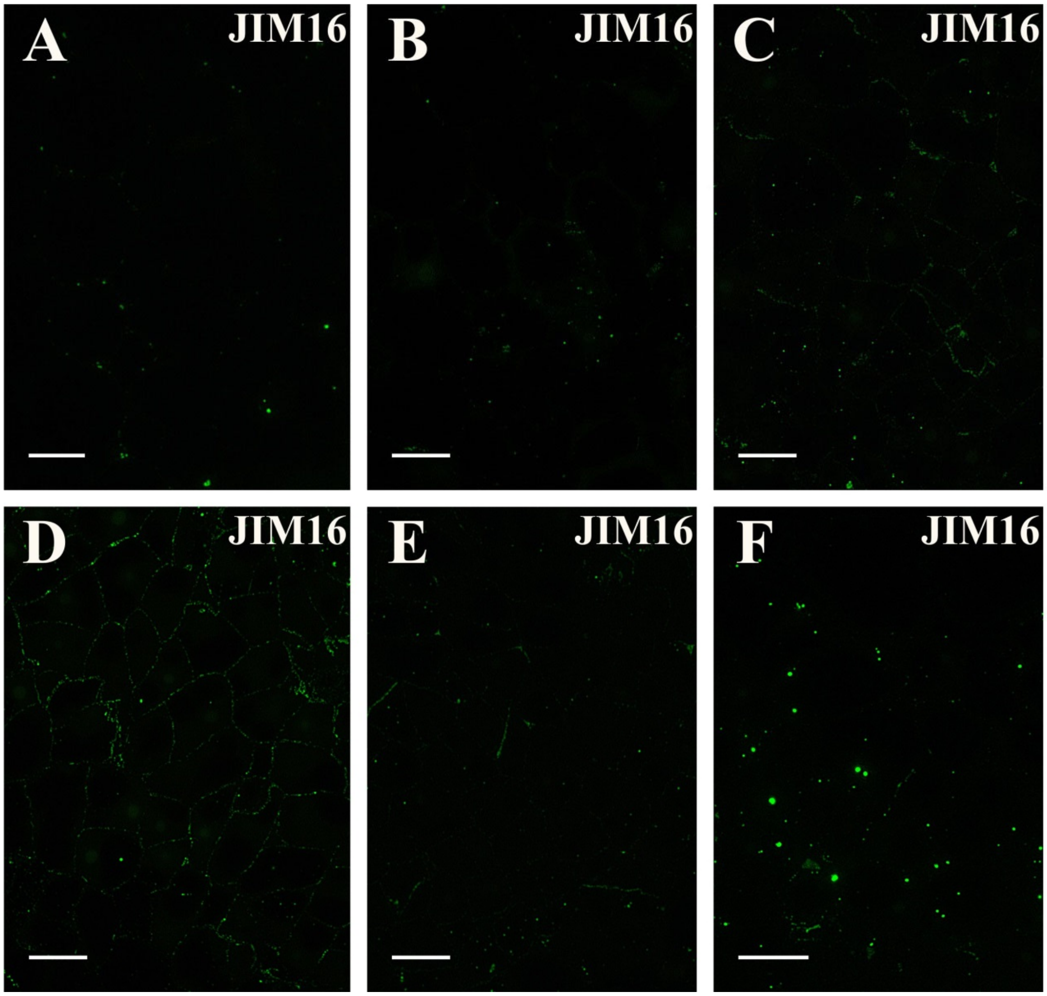

Figure S2I. The presence and distribution of the AGP epitope that is recognised by the JIM16 antibody (second repetition) was similar as described for the first repetition (Fig. 9). At the start of the culture (**A**) and after next days (**B** - two days; **C** - four days) signal was almost not detected. After five days (**D**) signal was detected in dividing cells (**C**), but in the following days of the culture the signal disappeared (**E** - ten days; **F** - 18 days). Scale bars: 10 μm.

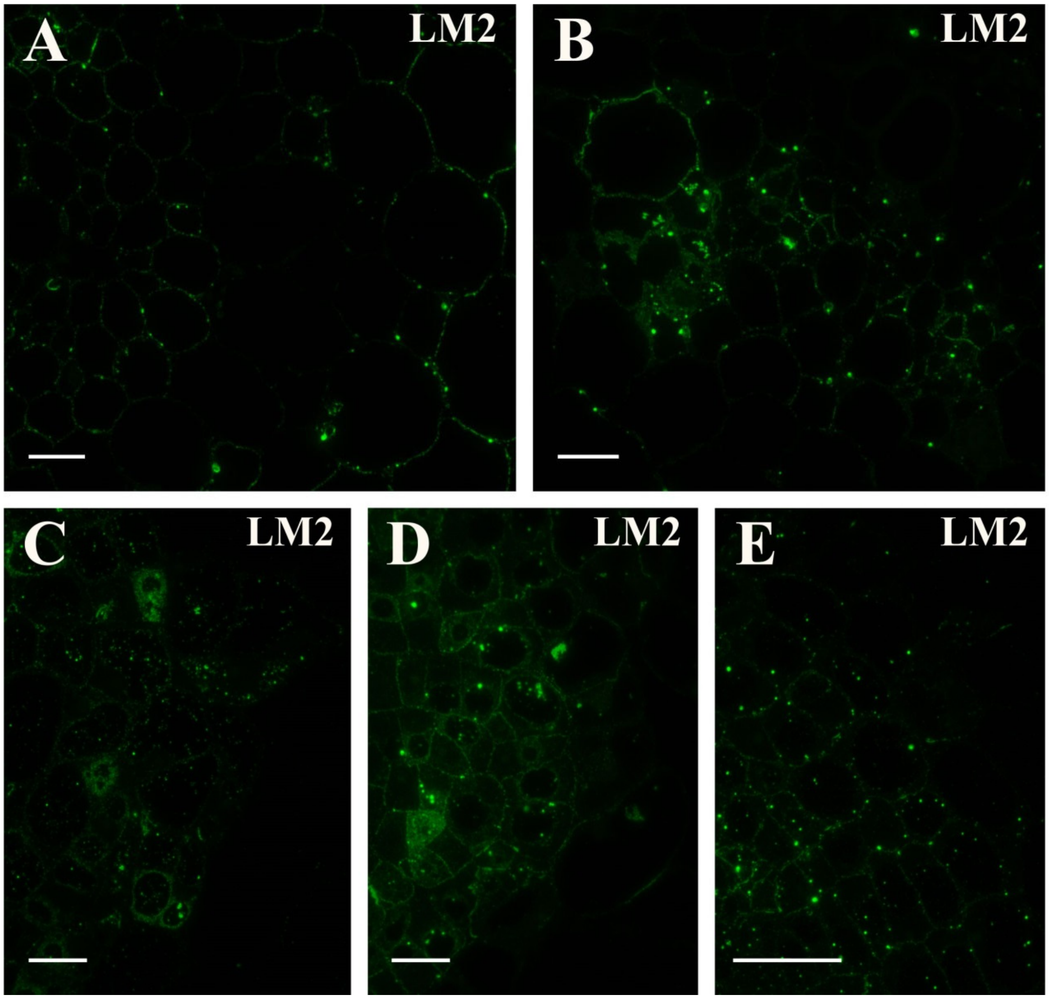

Figure S2J. Representative distribution of the AGP epitope that is recognised by the LM2 antibody obtained during the second repetition. (**A**) At the beginning of the culture characteristic lack of signal in pericycle cells located on the xylem pole was noticed. During the following days, the signal appeared in dividing cells (**B** – second day; **C** - forth day). At the end of the culture signal was still present in dividing cells (**D**), but successively disappeared in the explant surface cells (**E**). Scale bars: 10 μm.

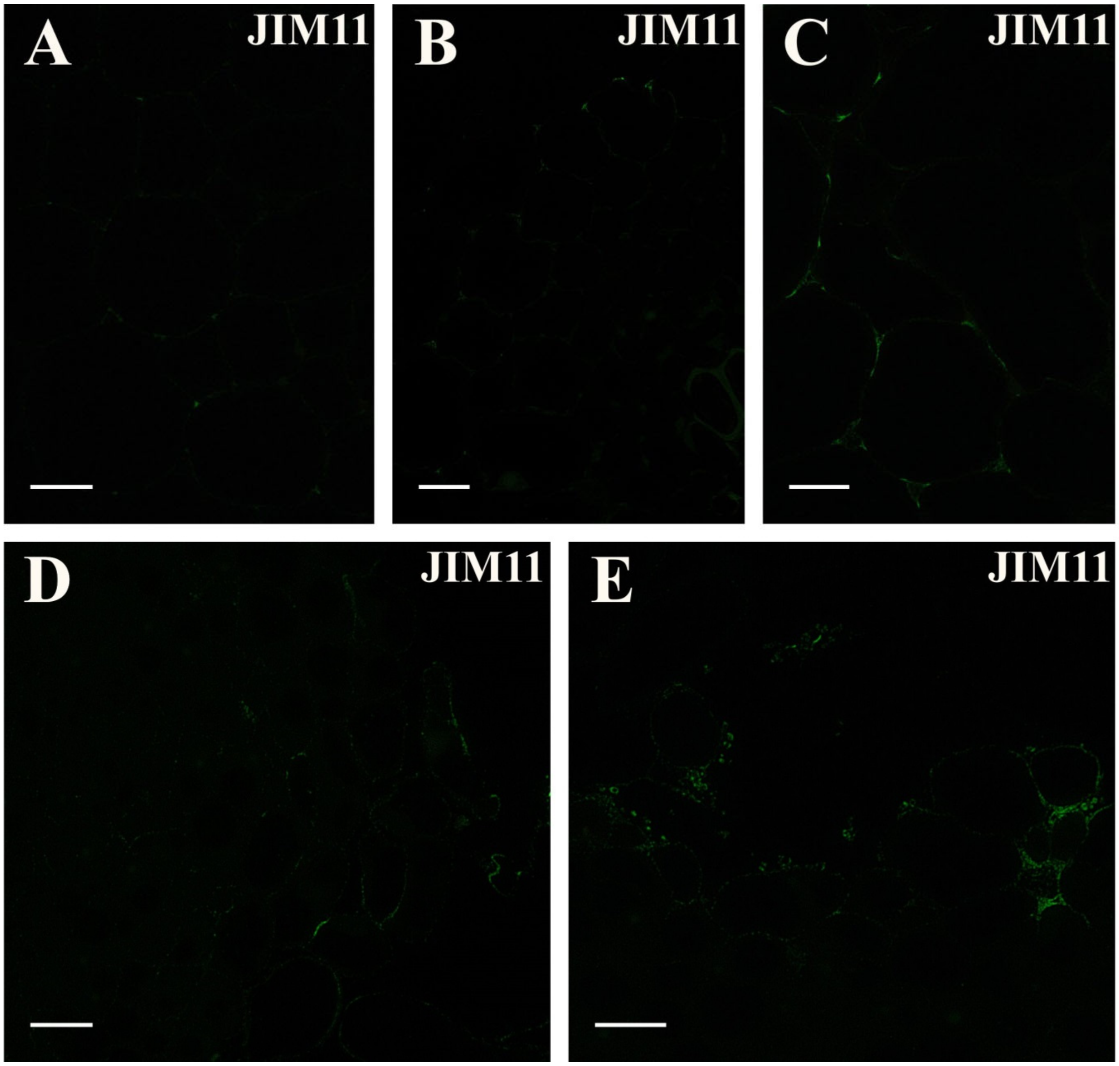

Figure S2K. Spatio-temporal distribution of the extensin epitope that is detected by the JIM11 antibody observed in explants during the second repetition. The epitope was mostly present in intercellular spaces which increased in size during the culture period [(**A**) – culture beginning, (**B**) - after two days, five days (**C**), ten days (**D**) and 18 days (**E**)]. Scale bars: 10 μm.

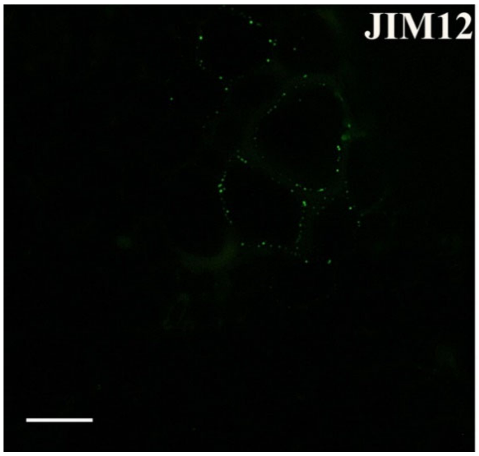

Figure S2L. The lack of extensin epitope that is detected by the JIM12 antibody in explant cells undergoing reprogramming (the second repetition). Scale bar: 10 µm.

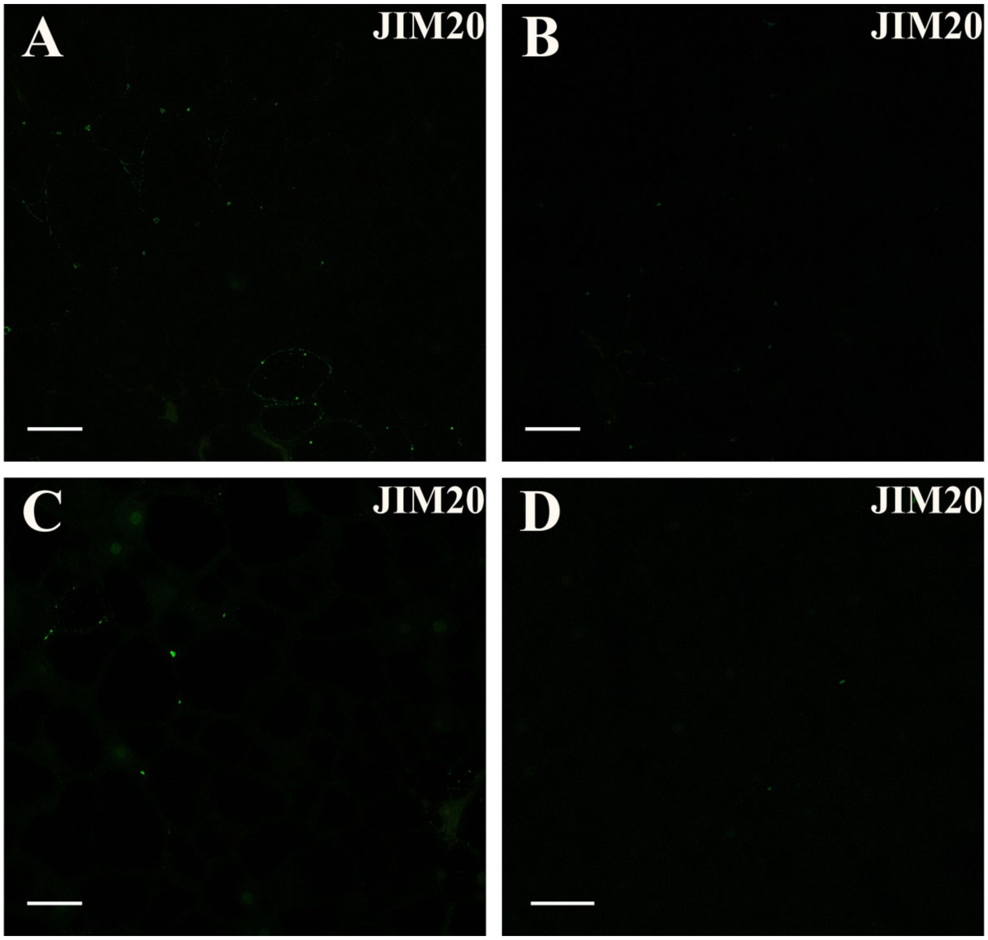

Figure S2M. Distribution of the extensin epitope that is detected by the JIM20 antibody observed during the second repetition was the same as is shown on Fig. 13 [(**A**) – start of the culture, after one day (**B**), five day (**C**), 10 days (**D**) of the culture. Scale bars: 10 µm.

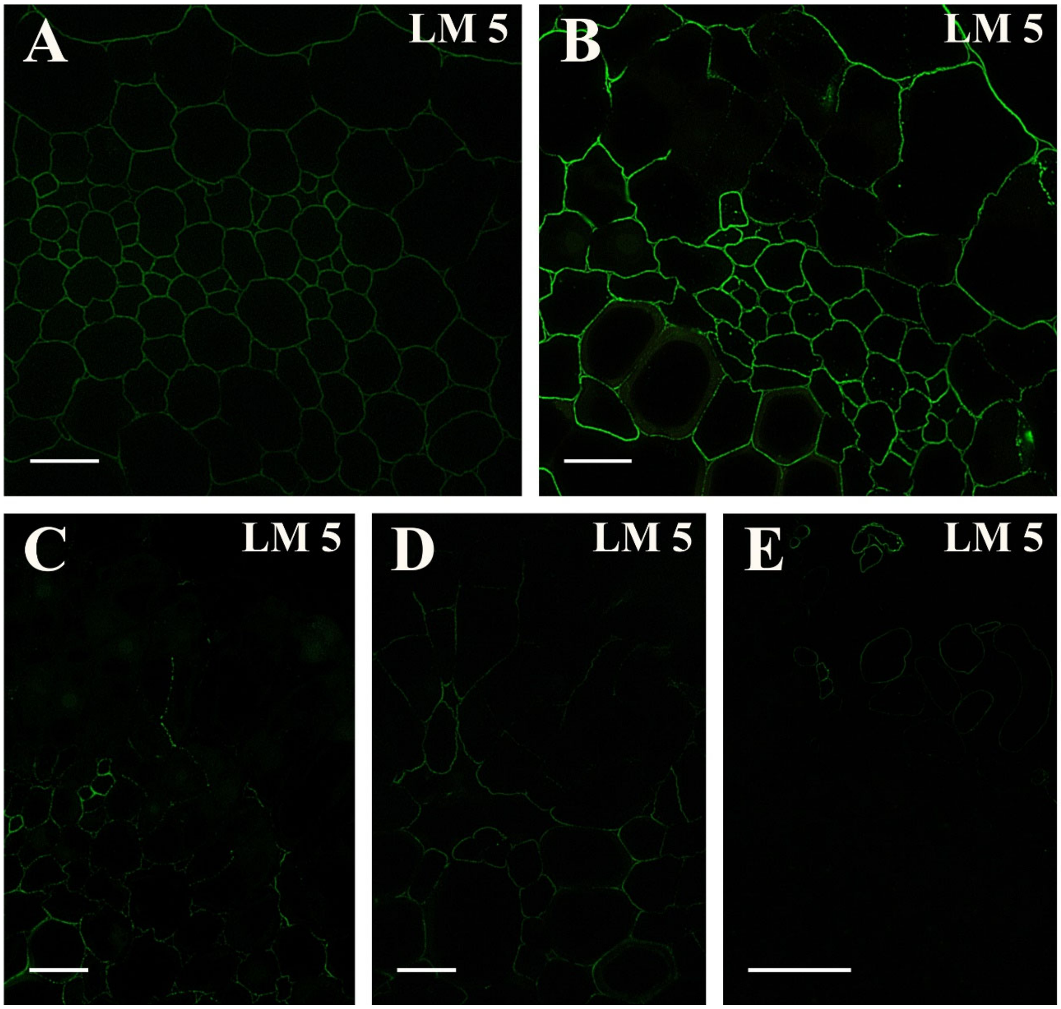

Figure S3A. During the third replication, analysis of the distribution of the pectic epitope that is recognised by the LM5 showed similar results to described for other replications [(**A**) – the beginning of the culture; (**B**) - after two days; (**C**) after four days; (**D**) - five days and 18 days (**E**) of the culture]. Scale bars: A-D – 10 μM; E – 50 μm.

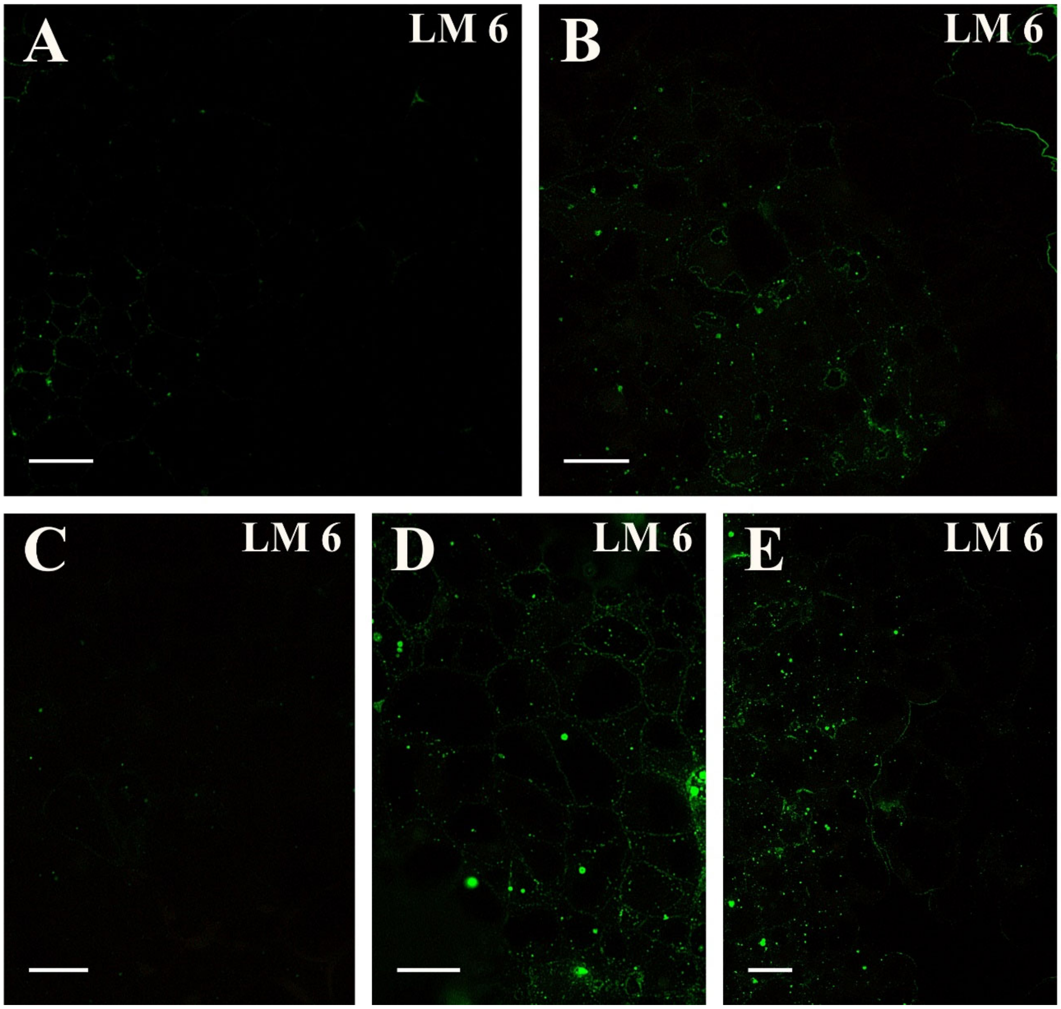

Figure S3B. Spatio-temporal distribution of the pectic epitope that is recognised by the LM6 antibody during following culture days obtained for the third repetition. (**A**) The signal was almost not detected at the beginning of the culture. After next days appearance of the signal in dividing cells (**B**), its disappearance at the fourth day (**C**) and re-appearance of the signal in the following days of the culture was noticed (**D** – fifth day; **E** - 18 days). Scale bars: 10 μm.

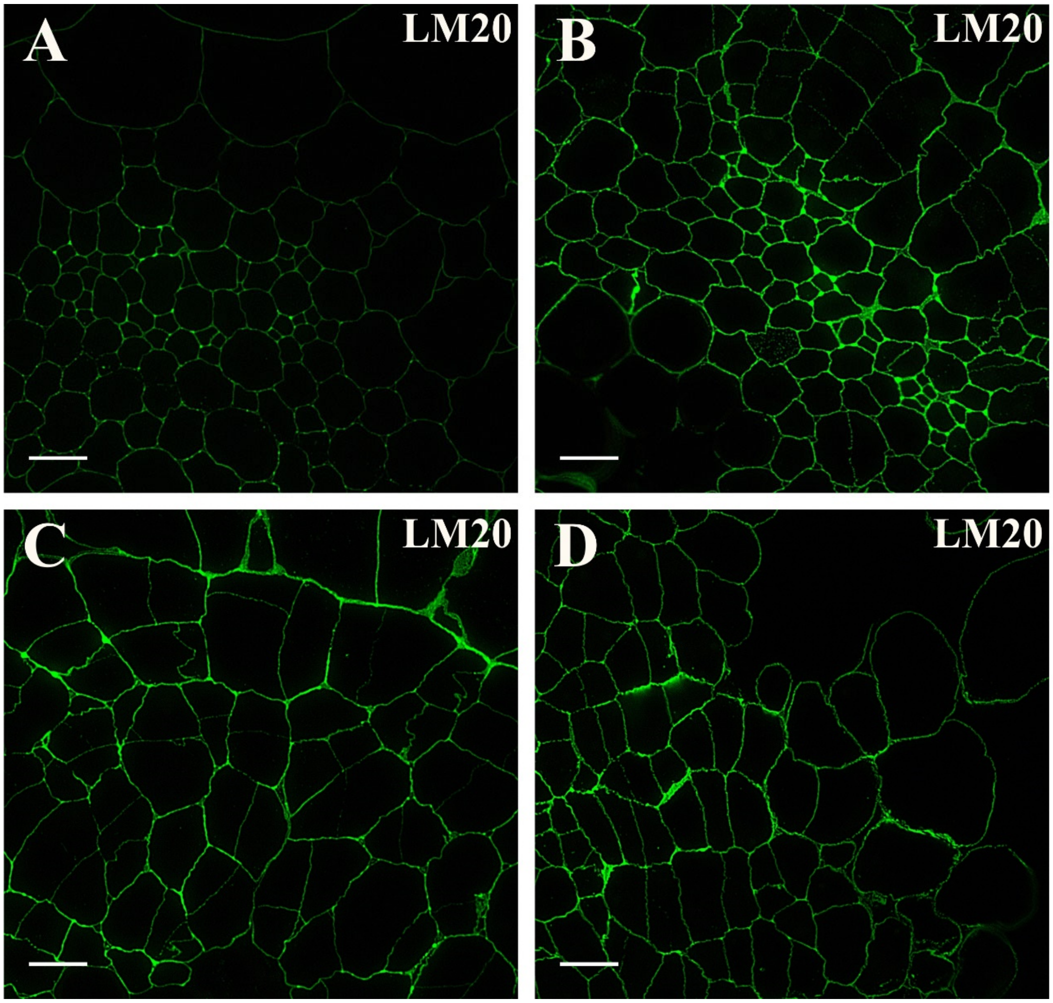

Figure S3C. Distribution of the pectic epitope that is recognised by the LM20 antibody observed during the third repetition was similar as described earlier (Fig. 3, Fig. S3/2). (**A**) Epitope distribution at the start of the culture. (**B**) Epitope presence after four days. (**C**) Localization of the epitope after five days and (**D**) 18 days of the culture. Scale bars: 10 μm.

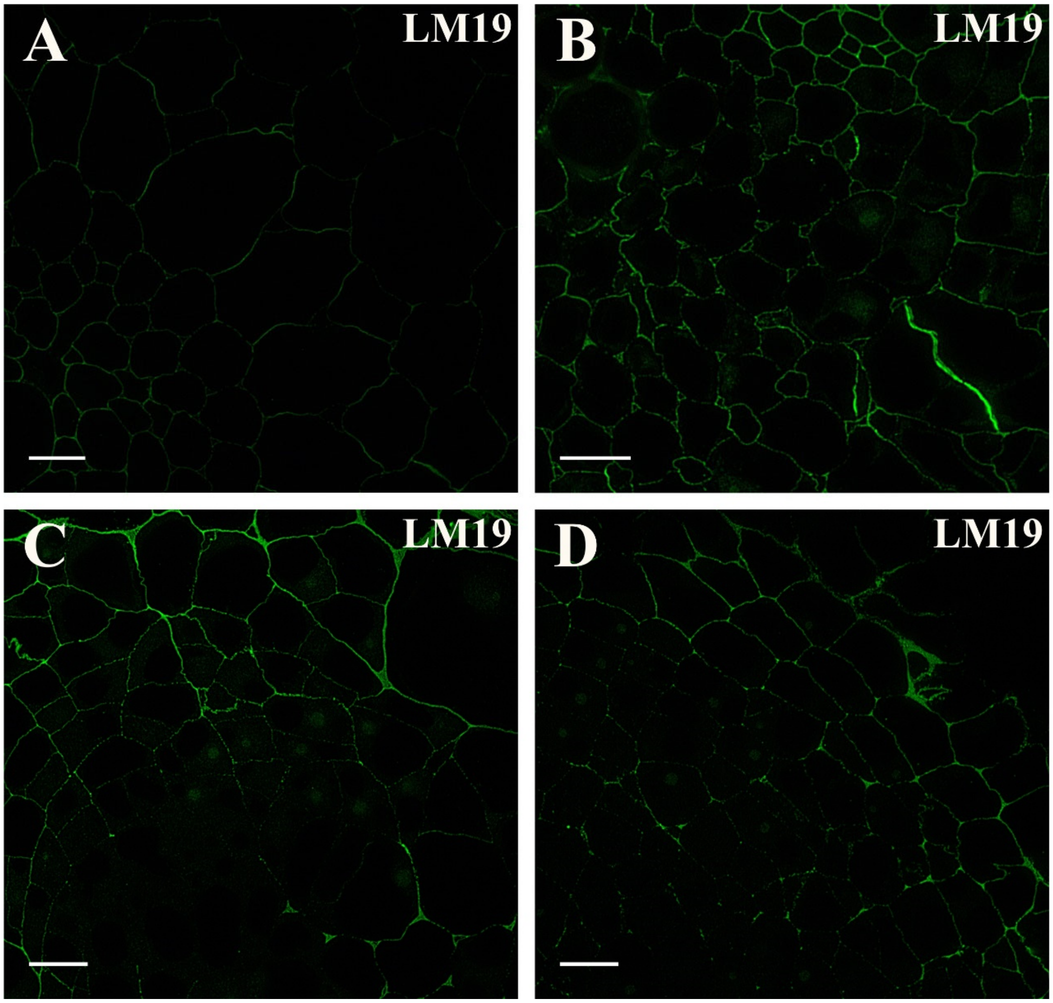

Figure S3D. Distribution of the pectic epitope that is recognised by the LM19 antibody observed in the analysis performed for the third replication. During the culture regardless of its duration epitope was detected in dividing cells as well as in intercellular spaces [(**A**) – start of the culture; (**B**) - after two days; (**C**) - four days and (**D**) - 18 days of the culture]. Scale bars: 10 µm.

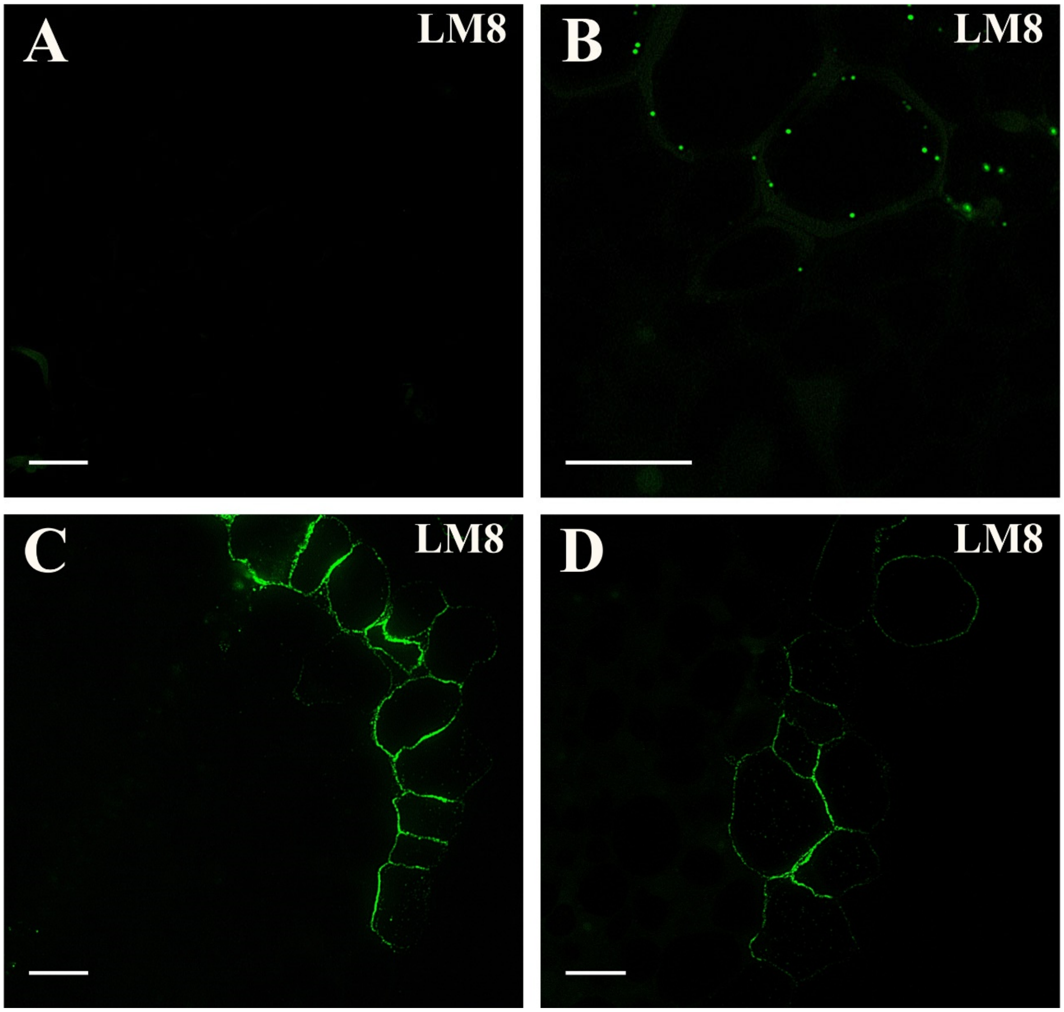

Figure S3E. Results of the analysis of the distribution of the pectic epitope that is recognised by the LM8 antibody confirmed the obtained results for other replications which have been carried out (Fig. 5, Fig. S5/2). Lack of signal was detected for the explant at the beginning of the culture (**A**). In next few days of the culture no important changes to the start of the culture was observed (**B**). Characteristic for cells predestined to separation (**C**) and separated (**D**) presence of the signal was well visible. Scale bars: 10 μm.

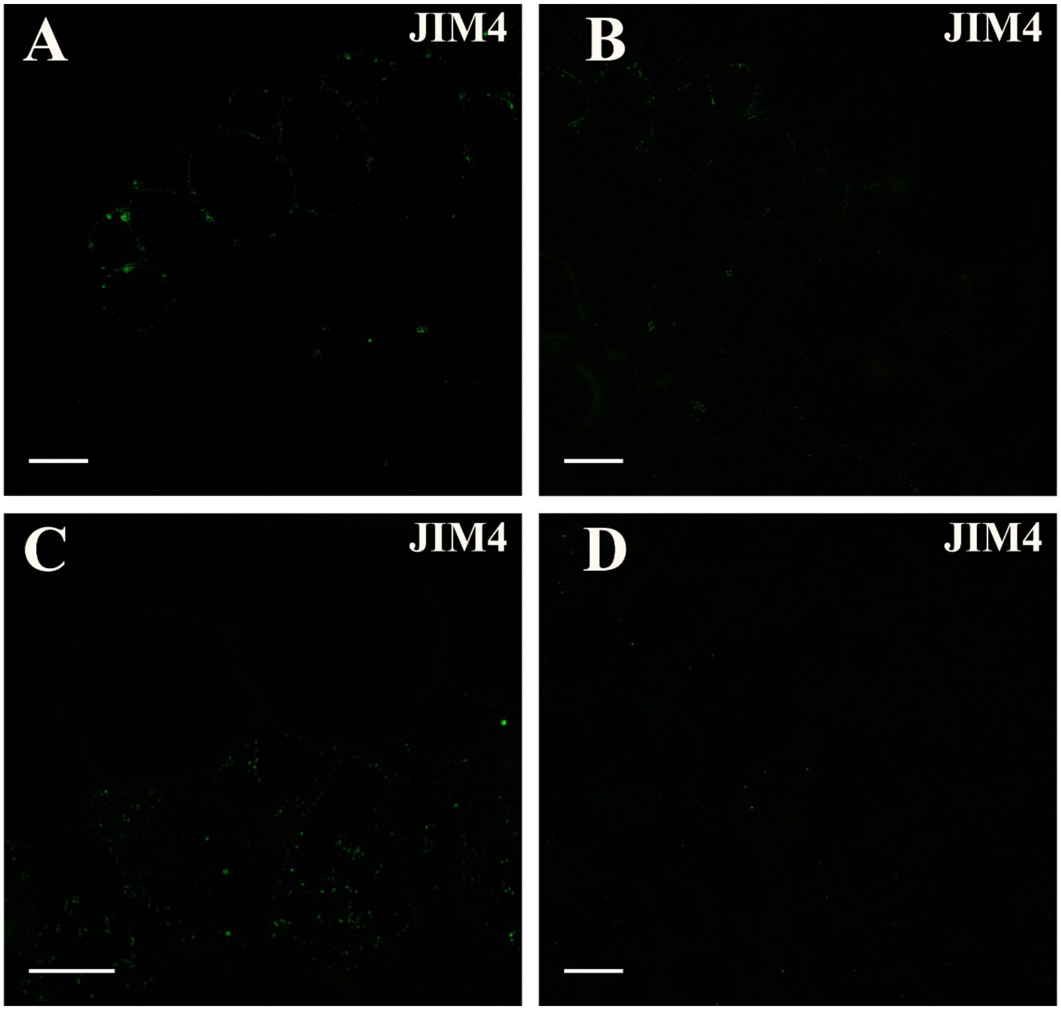

Figure S3F. The presence of the AGP epitope that is recognised by the JIM4 antibody during the analysis performed for the third repetition were consistent with results obtained for 1st and 2nd replication. Signal was hard to detect independent of the culture duration [(**A**) – start of the culture; (**B**) - after two days; (**C**) – after four days and (**D**) - 18 days]. Scale bars: 10 μm.

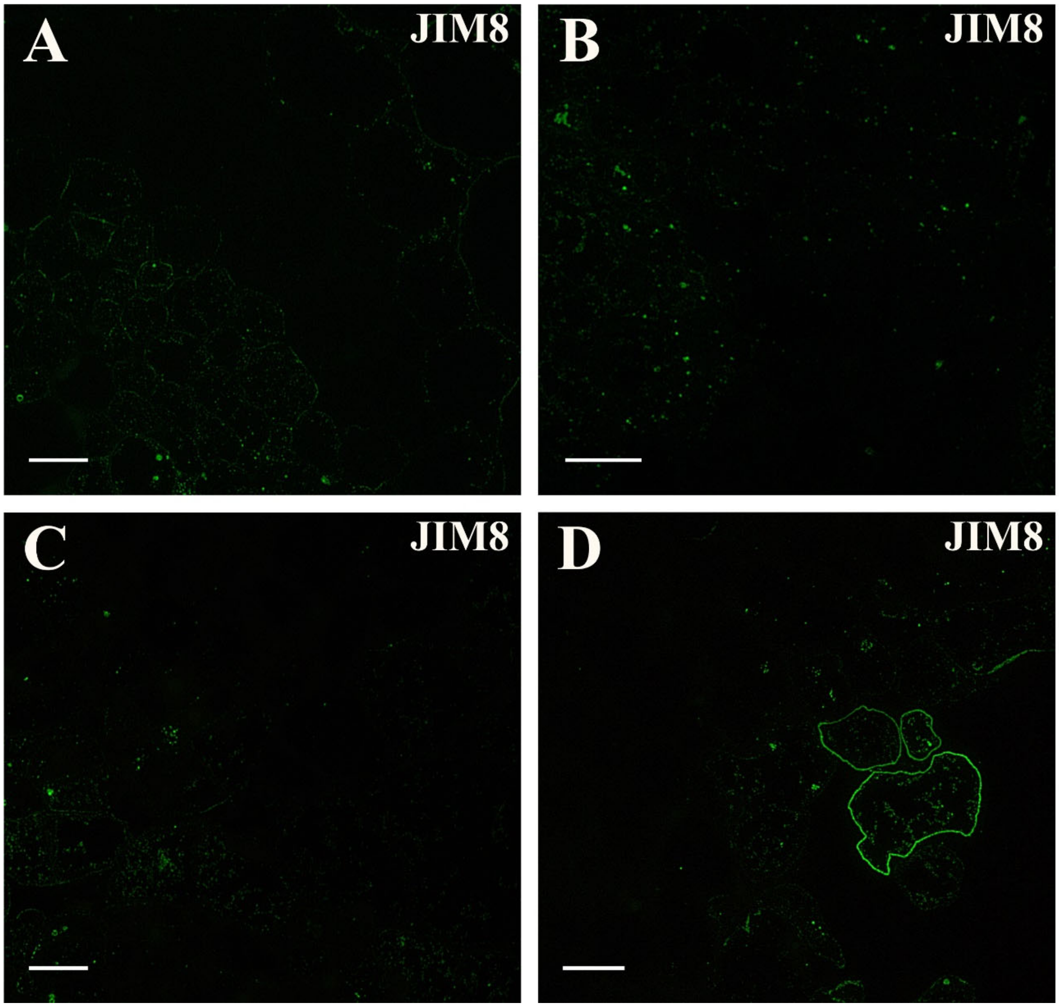

Figure S3G. Representative distribution of the AGP epitope that is recognised by the JIM8 antibody detected during the third repetition. At the beginning of the culture lack of signal marks the pericycle cells located on the xylem pole (**A**). In the following days of the culture signal was detected mostly in cytoplasmic compartments [(**B**) – third day; (**C**) - fifth day; (**D**) – 18th day]. Scale bars: 10 μm.

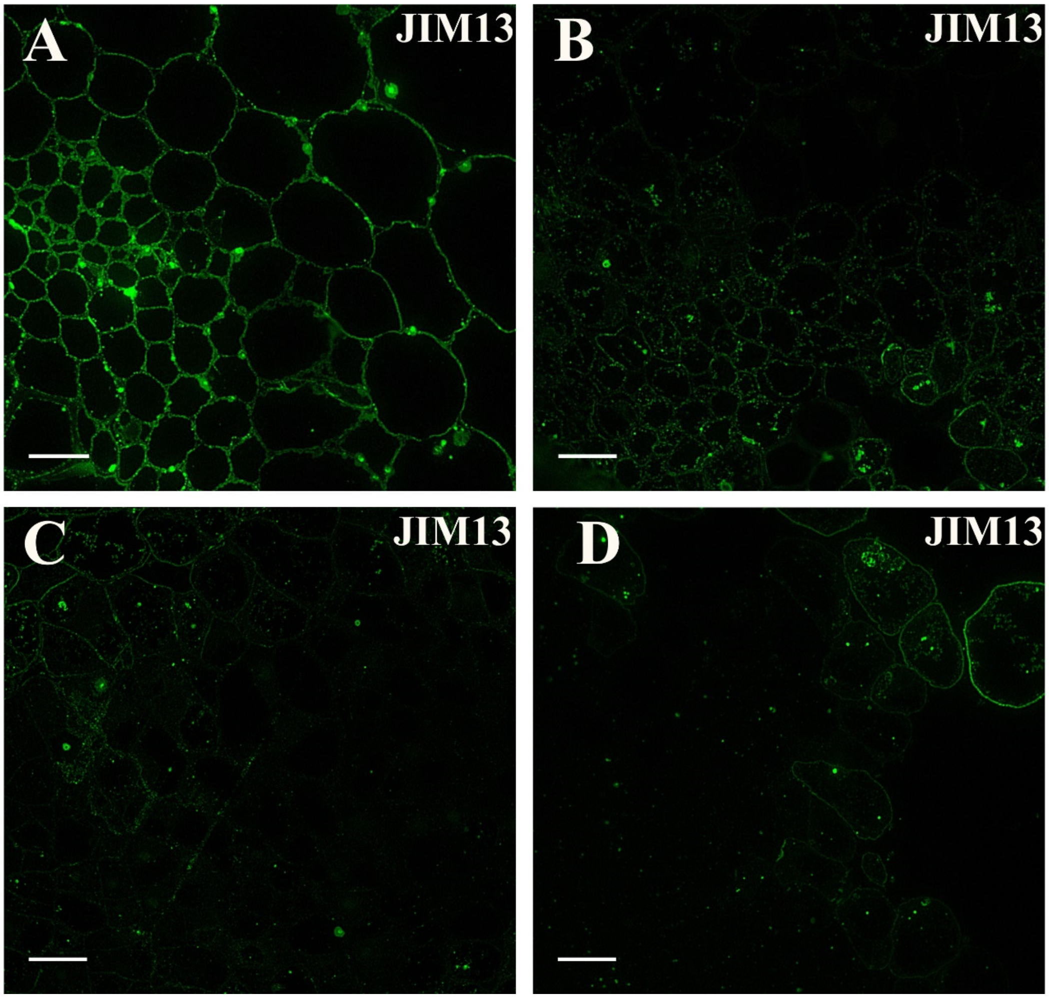

Figure S3H. The AGP epitope that is recognised by the JIM13 antibody was well visible at the beginning of the culture (**A**). During the first two days (**B**) lack of signal marks the pericycle cells from the xylem pole. (**C**) During following five days signal was present in dividing cells. (**D**) At the end of the culture clear signal was present in the separated cells but progressive loss of signal in the dividing cells was observed. All photos were obtained during the third repetition. Scale bars: 10 μm.

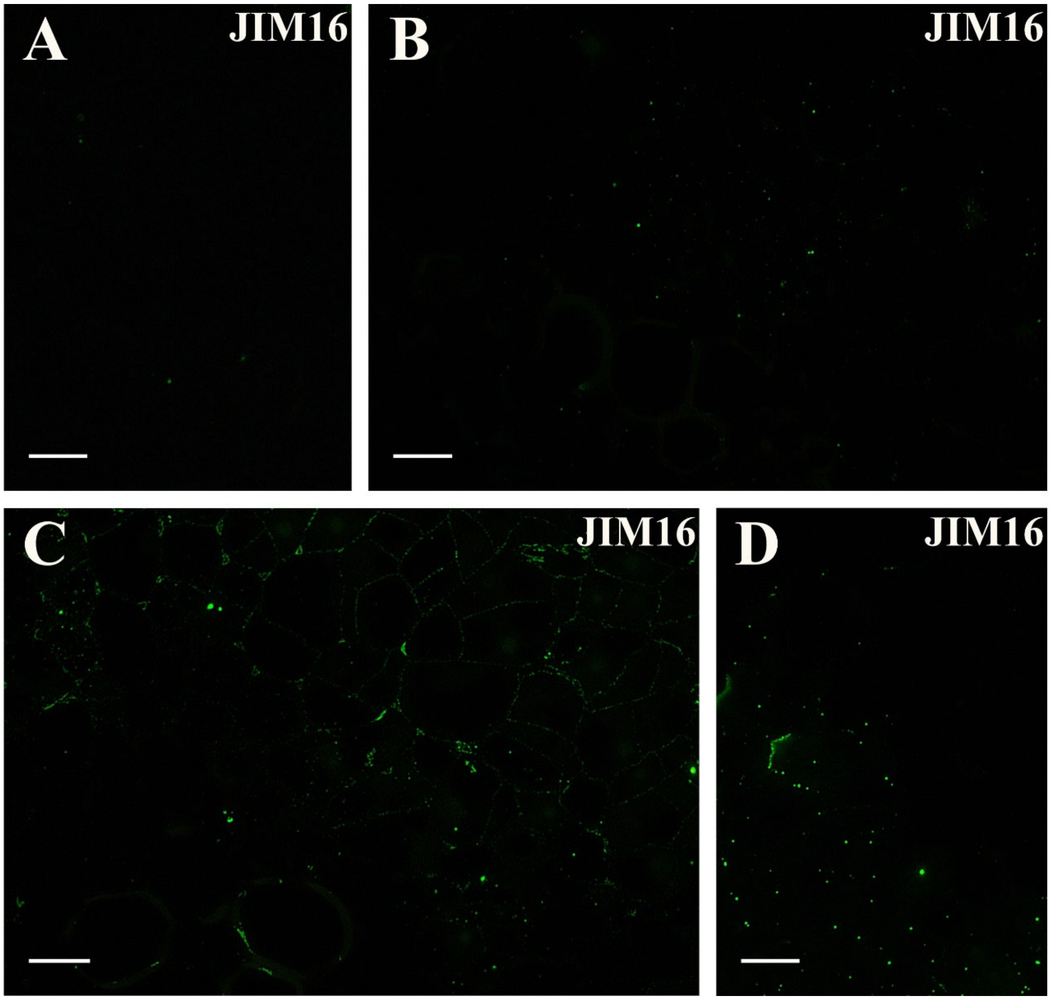

Figure S3I. Distribution of the AGP epitope that is recognised by the JIM16 antibody at the beginning (**A**), after two days (**B**), four days (**C**) and 18 days (**D**) of the culture detected during the analysis for third repetition. Obtained results are similar to obtained for other replications. Scale bars: 10 μm.

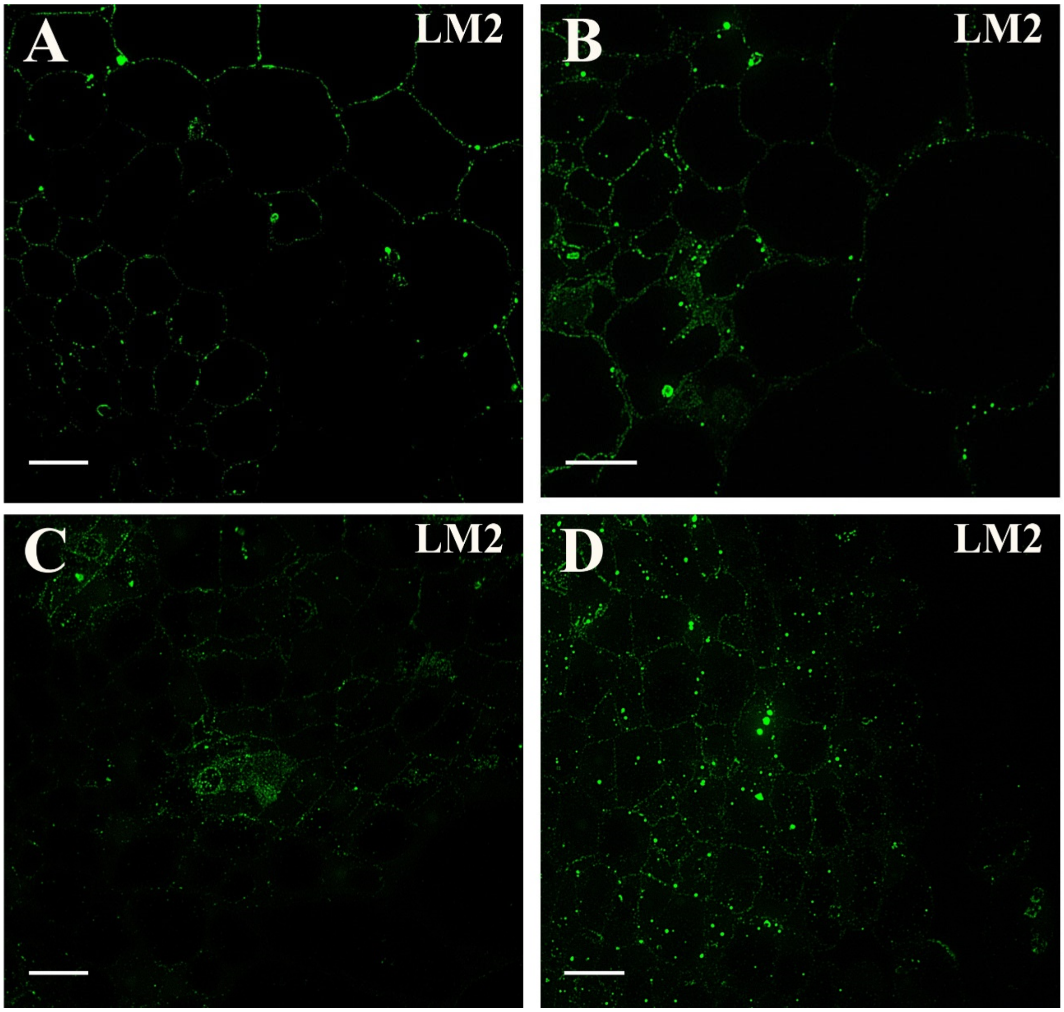

Figure S3J. Distribution of the AGP epitope that is recognised by the LM2 antibody (the third repetition). The analysed epitope was present in the cell walls and cytoplasmic compartment independent of the culture duration [(**A**) - start of the culture; (**B**) - after two days; (**C**) – after four days and (**D**) - 18 days]. At the beginning of the culture, similarly as in other performed replications, the pericycle cells from the xylem pole are marked by the lack of signal. Scale bars: 10 μm.

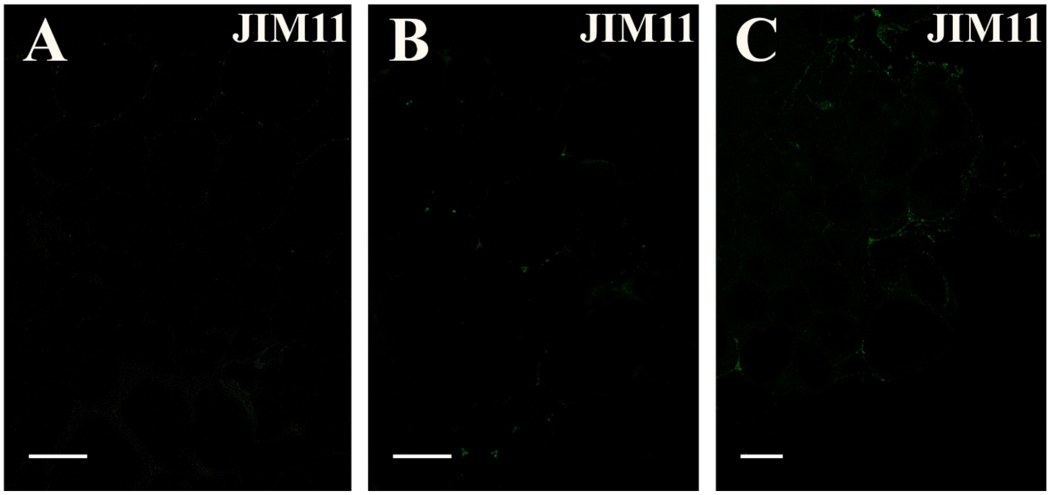

Figure S3K. The extensin epitope that is detected by the JIM11 antibody presence within the explant during the culture duration (analysis for the third replication) was similar to obtained for two other replications [(**A**) – culture beginning; (**B**) – fifth day; (**C**) – 18th day]. Scale bars: 10 μm.

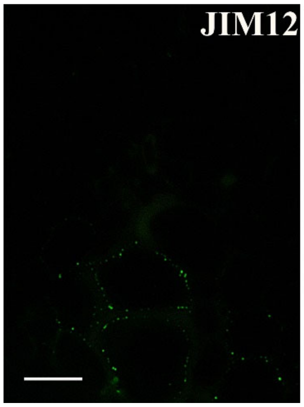

Figure S3L. Distribution of the extensin epitope that is detected by the JIM12 antibody observed during the analysis for the third replication. The photo is representative also for all other culture days. Scale bar: 10 μm.

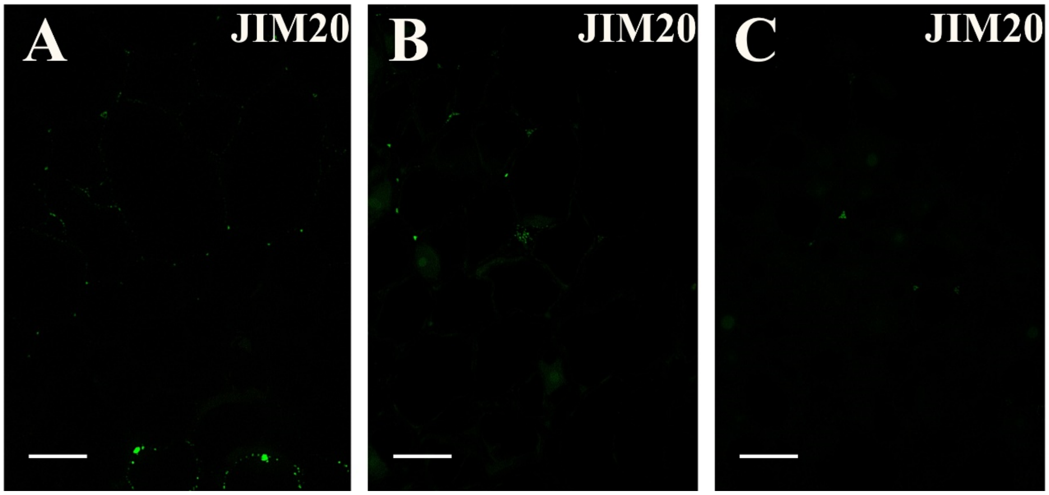

Figure S3M. Lack of the extensin epitope that is detected by the JIM20 antibody during the culture period (analysis for the third replication). [(**A**) – culture beginning; (**B**) - after five days and ten days (**C**) of the culture]. Presented results were obtained during the analysis for the third replication. Scale bars: 10 μm.
